# Long-term mortality of academy award winning actors and actresses

**DOI:** 10.1371/journal.pone.0266563

**Published:** 2022-04-13

**Authors:** Donald A. Redelmeier, Sheldon M. Singh

**Affiliations:** 1 Department of Medicine, University of Toronto, Toronto, Canada; 2 Evaluative Clinical Sciences Platform, Sunnybrook Research Institute, Toronto, Canada; 3 Institute for Clinical Evaluative Sciences in Ontario, Toronto, Canada; 4 Division of General Internal Medicine, Sunnybrook Health Sciences Centre, Toronto, Canada; 5 Centre for Leading Injury Prevention Practice Education & Research, Toronto, Canada; 6 Schulich Heart Program, Sunnybrook Health Sciences Centre, Toronto, Canada; Shantou University, CHINA

## Abstract

**Background:**

Social status gradients are powerful health determinants for individuals living in poverty. We tested whether winning an Academy award (Oscar) for acting was associated with long-term survival.

**Methods:**

We conducted a longitudinal cohort analysis of all actors and actresses nominated for an Academy award in a leading or a supporting role. For each, a control was identified based on age, sex, and co-staring in the same film.

**Results:**

Overall, 2,111 individuals were analyzed with 1,122 total deaths occurring during a median follow-up of 68.8 years. Comparisons of winners to controls yielded a 4.8% relative difference average life-span (95% confidence interval: 1.6 to 7.9, p = 0.004), a 5.1 year absolute increase in life expectancy (95% confidence interval: 3.0 to 7.2, p < 0.001), and a 41% improvement in mortality hazard (95% confidence interval: 19 to 68, p < 0.001). The increased survival tended to be greater in recent years, for individuals winning at a younger age, and among those with multiple wins. The increased survival replicated in secondary analyses comparing winners to nominees and was not observed in analyses comparing nominees to controls.

**Conclusions:**

Academy award winning actors and actresses show a positive association between success and survival, suggesting the importance of behavioral, psychological, or other modifiable health factors unrelated to poverty.

## Introduction

Social status is a widespread predictor of death and disease in diverse populations. The COVID-19 pandemic, for example, demonstrated a higher incidence and lethality among poor compared to wealthy adults living in the same region [1, 2]. Past literature suggests these health gradients may extend to intermediate levels of status and are not isolated to extremes of poverty or marginalized individuals [3, 4]. Our prior report of Academy award (Oscar) winning stars, in particular, found a 3.9 year greater life-expectancy compared to co-stars who did not win (79.7 vs 75.8, p = 0.003) [5]. These gradients in survival were not easily attributed to human genetics, environmental conditions, or access to care. Instead, the findings suggested the importance of behavioral or psychological factors in health [6–8].

The life-expectancy of Academy award winners has remained a topic of global attention, scrutiny, and debate [9, 10]. Twenty years have elapsed since the original report allowing now for larger sample size and more follow-up for estimating life-expectancy [11, 12]. One uncertainty focuses on immortal time bias (also termed survival bias) that postulates an unfair advantage for winners who might need to survive long enough to win and might yield a spurious life-expectancy estimate [13–15]. This is similar to a reverse-causality fallacy on whether winning leads to a longer life or whether a longer life leads to winning (social selection) [16–18]. Here we update earlier findings, address immortal time bias, and test whether Academy award winning actors and actresses live longer than less successful performers.

## Methods

### Selection of performers

Methods for identifying individuals are discussed in detail elsewhere, consistent with prior research, and reviewed here for clarity. Specifically, we identified every performer nominated for an Academy award (Oscar) for acting since inception (1929 to 2020). For each individual we also identified another cast member who performed in the same film as the nominee, with closest-possible matching on age and exact matching on gender. This approach ensured both performers were alive, working, prevailing in casting calls, succeeding in auditions, and winning good movie roles. This approach did not require that either performer survived to film distribution or subsequent recognition.

### Example of selection process

We provide an updated example of this selection process to illustrate the underlying technique. Meryl Streep was first nominated for a leading actress Academy award in 1988 for her performance in the film "Ironweed". Four other women were cast members in the same film as a leading actress or supporting actress, including Margaret Whitton. Meryl Streep was born in 1949, and Margaret Whitton was born in 1950; these two actresses had a 1 year difference in age. The other three actresses in the film all had an age difference greater than 1 year compared to Meryl Streep. Hence, Margaret Whitton was selected as the co-star for Meryl Streep in this film for this year.

### Details of selection process

We repeated the selection process for all years, sexes, and roles (leading or supporting). This process allowed one performer to have multiple co-stars over time; for example, the 2011 nomination for Meryl Streep was her 17th nomination. The selection process was also blinded to past or future events; for example, a 2003 nomination for Meryl Streep was linked to Tilda Swinton who subsequently won an Academy award in 2008. Co-stars overlapped in some cases; for example, Vivian Leigh and Kim Hunter won Academy awards for the same film in 1952 and were linked to the same control. No performer was counted more than once and no performer was dropped because of missing data.

### Documenting births and deaths

We determined each person’s date of birth and date of death by combining Internet and written sources. Conflicts were resolved by accepting information from printed sources over internet sources and conflicts between different internet sources were resolved by accepting information from the AllMovie database over other Internet sources [19]. No birth dates were missing; however, some birth dates were updated after the person’s death and we accepted information in the obituary as the most authoritative. These methods were similar to past research, including verifying individuals sometimes mistakenly rumored dead [20, 21]. People not known to be dead were assumed to be alive.

### Performer characteristics

Additional data were retrieved using analogous methods with an expanded number of sources. Data on country of birth, name changes after birth, year of first film, and main film genre were based on the AllMovie database. Data on pseudonyms were collected as a marker of public scrutiny. Data on race was determined by self-identification. Missing data were assumed to indicate the United States as the country of birth, dramatic film genre, no name change, and white racial group so no person was excluded from secondary subgroup analyses. Cause of death data were retrieved by inquiry to the National Film Information Service and contact with agents, with missing data listed explicitly. We did not collect data from Cannes, Golden Globes, or other award recognitions.

### Simple classification of social status

Many performers appeared multiple times because a career can include many different films. We counted each person only once and our initial approach simply classified performers according to highest achievement in acting. The three groups were winners (nominated and winning at least one Academy award), nominees (nominated but never winning) and controls (never nominated and never winning). The advantages of static classifications were that the groupings were easy to define, straightforward to analyze, and validated at follow-up. The disadvantage of static classifications was that this approach ignored changes in achievement over time and can result in misleading estimates [22, 23].

### Dynamic classification of social status

One innovation in our current analysis related to classifying each person’s status in a time-dependent manner based on accumulating achievement (S1 Appendix). This approach defined achievement as a dynamic factor that can change each year for the performer rather than staying as a static value throughout life [24]. For example, Meryl Streep was defined as a performer in 1977 following her first film, a nominee in 1978 following first nomination, and a winner in 1979 following first win. The advantage of dynamic classification using time-dependent statistical models was that the approach could subsequently account for the timing of an achievement, immortality bias, and other potential factors [25].

### Immortal time bias

A second nuance in dynamic time-dependent statistical models is that a large number of additional landmarks can help define a sustained career. In particular, an individual was not included until they had performed in at least one film. As a consequence, the date of a person’s first film is important since each individual must survive at least that long to be considered for analyses. For example, Meryl Streep co-stared with Susan Blommaert in 2009 for the film "Doubt" yet had a film debut one decade younger than Blommaert. An older starting age can introduce an immortality bias that exaggerates the survival of controls who co-star with individuals who have long successful careers [26].

### Delayed study enrollment

A related nuance is that an individual was not included in our study until they had performed in at least one film recognized by an Academy award nomination for at least one actor or actress. As a consequence, the age of a performer’s enrollment into the study is also important since each individual must generally survive at least that long to be included in analyses (and the lag between first film debut and actual enrollment can be many years). This means controls are not a representative sample of the general public and, instead, a select sample with sufficient talent, connections, and strength to become acquainted with celebrities. This selection yields a further immortality bias potentially exaggerating the survival of controls.

### Survival outcome

The sample size yielded opportunities for many different statistical estimates of survival. Life-span was the most straightforward and measured as the number of years between birth and death [27]. Life-expectancy was similar except that it added data from those who had not yet died (eg, survivorship methods adjusting for censoring related to those still alive) [28, 29]. Relative hazard rates were a third metric where differences in mortality rates were described as a relative differential combined from consecutive time points assuming no prior event [30]. The analysis of hazard rates with time-dependent covariates is the most standard statistical approach for clinical trials in medicine with limited follow-up [31].

### Statistical analysis

Congruent with past research, the primary analysis compared the survival of Academy award winners to controls and estimated life-expectancy from the survival curve. In addition, we developed multistate transition models to account for delayed film debut, delayed study enrolment, and birth era using the bootstrap approach to test differences (S1 Appendix). All major secondary analyses were replicated including exploring the number of wins and age at first win. Analyses were conducted using Python software 3.8 version (Python Software Foundation, Fredericksburg, Virginia) and validated using SAS software 9.4 version (SAS Institute, Inc., Carey North Carolina). The study was approved by the Sunnybrook Research Ethics Board. All p-values are two tailed.

Multistate transition models are an extension of survival analysis that include intermediate states between a person’s initial state and final state [32, 33]. Our multistate model included four intermediate states between birth (initial state) and death (final state) that accounted for incomplete data on survival (right-censoring), delayed study enrollment (left-truncation), and changes in status (S1 Appendix). The model directly accounted for immortal time and was further expanded to partially account for demographics (birth year, gender), healthy performer bias, and posthumous recognition (S1 Appendix) [34]. Multistate model results were also calculated as absolute survival differences for comprehensive adjustments.

Three assumptions were used to handle data anomalies for multistate models. First, we discounted posthumous events so status terminated at the time of death; for example, follow-up on Heath Ledger ended with his death in 2008 rather than his win in 2009. Second, we identified multiple transitions in the same year by counting only the highest; for example, Lupita Nyong’o was first nominated in 2013 and won the same year thereby contributing no years as a nominee and all subsequent years as a winner. Third, we considered time in one-year intervals thereby ignoring the exact day of death or month of an award ceremony. These assumptions were restricted to the multisite model to simplify computer coding.

We replicated analytic comparisons using the proportional hazards model as an added test of robustness. These models included a time-dependent step-function to account for age at first film, age at study enrollment, age at first nomination, and age at first win. The purpose was to estimate relative hazard rates (not absolute differences of life-expectancy) while controlling for multiple confounders simultaneously (eg, birth year, sex, race). The limitation of such models is the semi-parametric proportionality assumption that is difficult to test fully with time-dependent factors [35]. Results were expressed as inverse hazard rate ratios where values greater than 1.00 indicated increased survival.

## Results

### Overview

A total of 2,111 performers were nominated for an Academy award or appeared opposite the nominated performer since inception in 1929. The characteristics of winners, nominees, and controls were similar in birth year, sex, race, country of birth, and film genre (Table 1). The mean age at first film was 27.0 years, with significantly earlier ages for winners relative to controls (p < 0.001 for each comparison). The mean age at first nomination was 38.5 years with no significant difference for winners relative to nominees (p = 0.659). The mean age at first award win was 41.5 years, most who won received an award before age 50 years (n = 235, 77%), and few had multiple wins (n = 45, 15%).

**Table 1:** Baseline characteristics *.

		Winners †	Nominees †	Controls †
		(n = 305)	(n = 629)	(n = 1177)
Birth year	before 1900	34 (11)	79 (13)	149 (13)
	1900–1919	78 (26)	96 (15)	252 (21)
	1920–1939	67 (22)	182 (29)	288 (24)
	1940–1959	55 (18)	150 (24)	225 (19)
	1960–1979	60 (20)	87 (14)	201 (17)
	1980–1999	11 (4)	34 (5)	62 (5)
	2000–2019	0 (0)	1 (0)	0 (0)
Sex	Male	152 (50)	317 (50)	640 (54)
	Female	153 (50)	312 (50)	537 (46)
Race	White	288 (94)	583 (93)	1107 (94)
	Non-white	17 (6)	46 (7)	70 (6)
Birth Country	United States	205 (67)	423 (67)	819 (70)
	International	100 (33)	206 (33)	358 (30)
Name	Changed	69 (23)	127 (20)	94 (8)
	Unchanged	236 (77)	502 (80)	1083 (92)
Film Genre	Drama	236 (77)	468 (74)	799 (68)
	Any other	69 (23)	161 (26)	378 (32)
Age at first film (years)	Mean ±std dev	25.5 ± 8.2	25.5 ± 9.2	28.3 ± 9.8
Age at enrollment (years)	Mean ±std dev	37.3 ± 12.1	37.8 ± 13.5	41.3 ± 12.9

* all data as percentages, except where noted as years.

† updated status defined on July 1, 2020.

### Observed life span

Overall, 1,122 performers had died by July 1, 2020 (mean follow-up from birth of 68.8 years). The average age at death for winners was 77.1 years, for nominees was 73.7 years, and for controls was 73.6 years. A t-test comparing winners to controls yielded a 3.5 year absolute difference in average life-span (95% confidence interval: 1.2 to 5.8). This simple comparison equaled a 4.8% relative increase in life years (95% confidence interval: 1.6 to 7.9). Similar calculations comparing winners to nominees yielded a 3.4 year absolute difference in average life-span (95% confidence interval: 0.8 to 6.1) equal to a 4.6% relative increase in life years (95% confidence interval: 1.1 to 8.2).

### Causes of death

A cause of death was identified for 959 individuals and missing for 163 additional individuals (Table 2). An identified cause of death was more frequent for winners than controls (92.6% vs. 84.4%, p = 0.008) and similarly frequent for winners and nominees (92.6% vs. 93.5%, p = 0.723). No major imbalances were observed among the three groups in identified causes of death. Ischemic heart disease (n = 261, 23.3%) and malignant cancer (n = 294, 26.2% were the most common identified causes and accounted for half the total deaths. Most deaths (n = 1,057, 94%) occurred after age 50 years, more than a decade after their first film (n = 1,109, 99%), and more than a decade after enrollment (n = 1,009, 90%).

**Table 2:** Causes of death *.

	Winners †	Nominees †	Controls †
Ischemic Heart Disease	45 (30)	68 (21)	148 (23)
Cerebrovascular Disease	10(7)	27 (8)	37 (6)
Other Cardiovascular Disease	2 (1)	8 (2)	22 (3)
Malignancy	42 (28)	87 (27)	165 (25)
Chronic Lung Disease	7 (5)	12 (4)	20 (3)
Acute Pneumonia	14 (9)	21 (7)	30 (5)
Liver Failure	0 (0)	6 (2)	5 (1)
Kidney Failure	2 (1)	8 (2)	7 (1)
Primary Neurologic Disorder	3 (2)	15 (5)	16 (2)
Injury and Poisoning	6 (4)	26 (8)	30 (5)
Other Specific Cause	7 (5)	24 (7)	39 (6)
Unlisted Cause §	11 (7)	21 (7)	131 (20)
TOTAL	149 (100)	323 (100)	650 (100)

* all data as counts (percentage).

† updated status defined on July 1, 2020.

§ includes partial data (eg. "died of natural causes").

### Estimated life-expectancy

**Fig 1:**
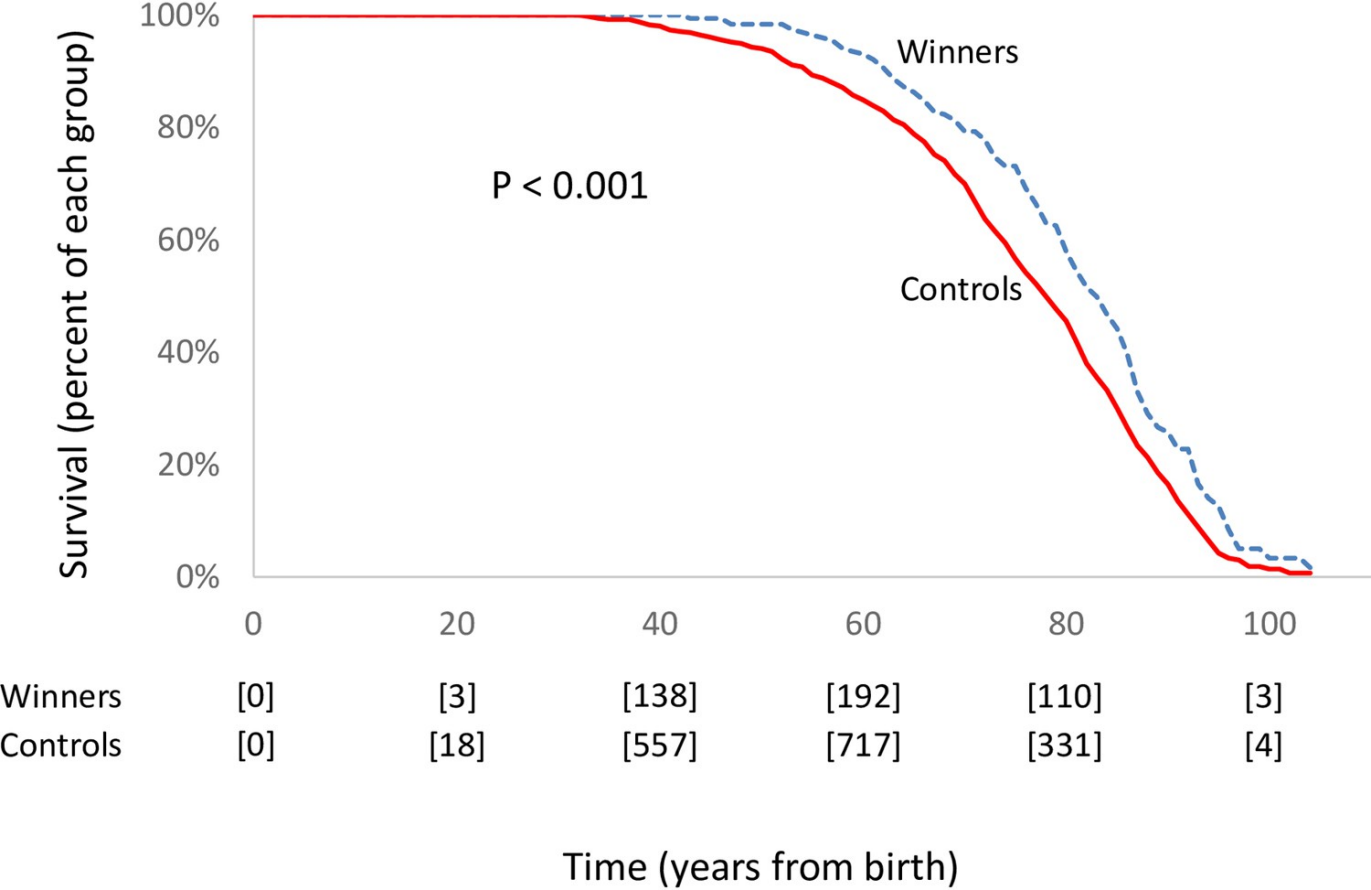
Survival of winners and controls. Survival plot comparing mortality between winners and controls based on multistate model. X-axis denotes time in years following birth. Y-axis denotes percent in each group surviving to corresponding time point. Winners as dashed blue line and controls as solid red line. P-value based on bootstrap statistic. Counts in square brackets indicate number of individuals in risk set at corresponding time point. Results show generally long life-expectancy and significant difference comparing winners to controls. Median survival for winners is 83 years and controls is 78 years.

Life-expectancy was estimated as survival through a multistate model to account for those who had not yet died. The life-expectancy for winners was 81.3 years, for nominees was 76.4 years, and for controls was 76.2 years (Fig 1). Comparisons of winners to controls yielded a 5.1 year absolute difference in life-expectancy (95% confidence interval: 3.0 to 7.2). This comparison equaled a 6.7% relative increase (95% confidence interval: 3.9 to 9.4). Similar calculations comparing winners to nominees yielded a 4.9 year absolute difference in life-expectancy (95% confidence interval: 1.9 to 8.3) equal to a 6.4% relative increase (95% confidence interval: 2.9 to 10.1).

### Additional predictors

Life-expectancy was also related to demographic characteristics. Those born in the recent era (after 1920) had a 6.0 year longer life-expectancy than those born in the remote era (S1 Appendix). Women had a 3.2 year longer life-expectancy than men. Identifying as non-white was associated with a 1.0 year shorter life-expectancy than identifying as white, yet the difference was not statistically significant. Birth country, acting genre, and changing names were not significant predictors of life-expectancy. Overall, a white actress in the recent era who won an Academy award had more than a 20 year longer life-expectancy than a non-white actor in the remote era who did not win (88.2 vs 65.6, respectively).

### Secondary analyses

**Fig 2:**
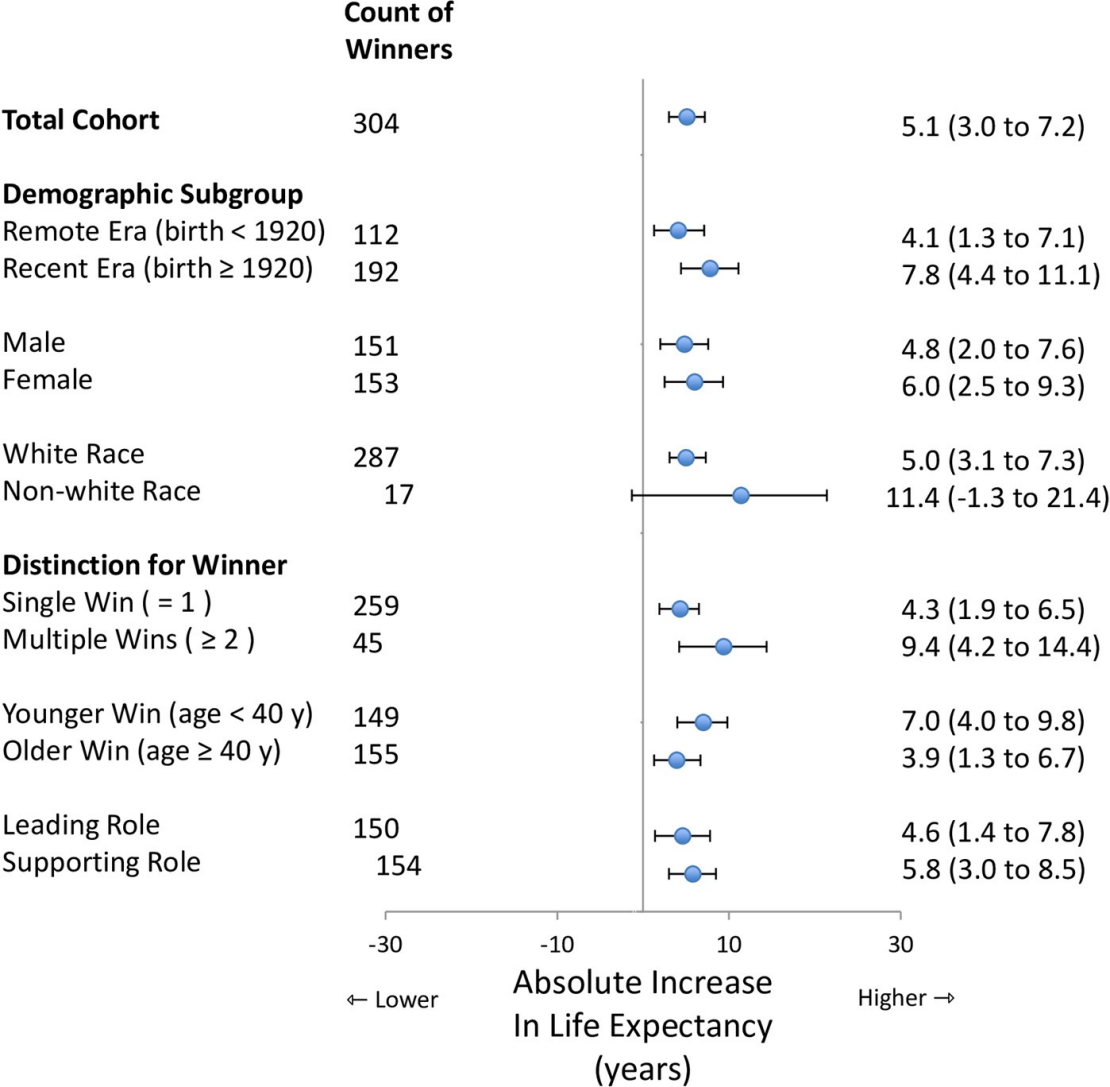
Secondary analysis of winners. Forest plot showing multiple sensitivity analyses of absolute increase in life-expectancy for winners in different subgroups. Referent group in all analyses is control performers with no win and no nomination. Circles denote estimate and horizontal lines denote 95% confidence interval. Vertical line for null association marked as a 0 year increase in life expectancy. Sample size in each analysis shown as corresponding count of winners. Findings suggest increased life-expectancy for winners compared to controls.

Not all Academy awards had identical implications for increased life-expectancy. The increase tended to be greater in recent eras yet still significant in remote eras (Fig 2). The increase tended to be greater for those with multiple wins yet still significant for those with a single win. The increase tended to be greater for those winning at a younger age yet still significant for those winning at an older age. The increase was similar for men and for women as well as similar for those in leading roles and those in supporting roles. In contrast, no significant increase in life-expectancy was evident for individuals with a single nomination or individuals with multiple nominations who did not win (S1 Appendix).

### Relative survival rates

Relative survival rate ratios were estimated using the proportional hazard model for the full cohort taking into account time-dependent covariates for an award nomination and an award win. The basic model suggested a 41% relative increase in survival rate ratio associated with winning (Table 3). As expected, birth era and sex were significant predictors whereas birth country, changing names and acting genre were not significant predictors of survival. Adjusting for baseline differences yielded a 41% relative increase in survival rate ratio associated with winning. No significant increase in survival was associated with an Academy award nomination as tested in adjusted analyses.

**Table 3:** Hazard ratios from proportional hazards analysis *.

		Basic Analysis ¥		Adjusted Analysis π	
		Rate	95% Confidence	Rate	95% Confidence
		Ratio †	Interval	Ratio †	Interval
Social status	Winner μ	1.41	1.18 to 1.67	1.41	1.19 to 1.68
Recognition	Nominee μ	1.18	1.04 to 1.34	1.10	0.97 to 1.25
Birth era	Recent	1.83	1.63 to 2.06	1.85	1.65 to 2.08
Sex	Female	1.36	1.21 to 1.52	1.33	1.19 to 1.50
Race	Non-white	0.93	0.66 to 1.31	0.79	0.58 to 1.07
Birth country	Non-USA	1.07	0.94 to 1.21	1.08	0.95 to 1.22
Name	Changed	1.03	0.90 to 1.19	0.98	0.85 to 1.14
Film genre	Drama	1.10	0.97 to 1.25	1.07	0.94 to 1.21

* estimates from hazards analysis of full cohort.

† values > 1.00 indicates longer survival (inverse hazard).

¥ no adjustment for other differences.

π adjusted for all baseline differences.

μ time dependent variable.

## Discussion

We examined over two thousand actors and actresses for over 100,000 life-years of follow-up to test the association between success and survival. We found that Academy award winners live significantly longer than their co-stars. The analysis replicated earlier findings from decades ago, showed a larger difference in life-expectancy than originally reported, and suggested the increased survival extends to analyses restricted to winners and nominees. The increased life-expectancy was greater for individuals winning in recent years, at a younger age, and with multiple wins. For context, a five-year difference in life-expectancy associated with an Academy award exceeds the magnitude of lost life-expectancy for the general US population associated with the COVID-19 pandemic [36, 37].

An important weakness in our study relates to the distinction between prognosis and causality. An inanimate statuette may foreshadow longer survival yet it is not a magical charm that directly improves a recipient’s health. Correlation does not mean causality any more than the link between female gender and longer survival would indicate a gender transition might increase an average man’s lifespan [38]. Instead, genetic features, environmental factors, personal attributes, or other unmeasured pathways remain topics for future research on longevity [39–41]. Similarly, the prognostic value of an Academy award can be descriptively accurate regardless of hidden variables [42]. Statistical modeling is no substitute for collecting more data exploring mechanisms driving an observed association.

One behavioral interpretation is that social status can contribute to health in celebrities and thereby may be important more widely in society. Successful actors often have personal chefs, trainers, chauffeurs, nannies, managers, coaches, and other staff who make it easier to follow a healthy lifestyle. Academy award winners are also surrounded by people interested in their well-being, invested in their reputation, empowered to enforce standards, and motivated to avoid scandals. The result may be that winners tend to eat properly, exercise consistently, sleep regularly, avoid drug misuse, and follow the ideals of a prudent life-style that bring more gains with adherence [43, 44]. These behavioral mechanisms suggest social gradients in disease might be mitigated by interventions for a healthy lifestyle.

Alternatively, several psychological mechanisms could expand on the biomedical model of disease by highlighting the social determinants of illness [45]. Academy award winners, for example, may be able to avoid some stress through more control and less aggravation when encountering an obstacle. The award, in particular, could soften a humiliating rejection or insulting review by preserving peace-of-mind and helping to buffer the hypothalamic-pituitary stress responses [46]. A related factor could be the subjective peace-of-mind and reduction in allostatic load that contributes to preserved telomere length, endothelial function, or neuroendocrine metabolism [47]. These diverse physiologic pathways and biomarkers remain topics for future research [48, 49].

Other explanations could include the adverse consequences when success is not achieved [50–52]. One possibility is lower resilience or another ingrained attribute that contributes to less success and less health [53]. A different factor might be the distraction of unfinished business that lingers in the background independent of acute stress (Zeigarnik effect) [54]. A third explanation might be unfulfilled ambition that leads to detrimental workaholism [55]. A more interactive mechanism may involve social networks where award winners cultivate a different set of friendships relative to others in the movie industry [56, 57]. None of these competing theories is an easily testable hypothesis, a readily modified feature, or a reason why life-expectancy observe in all groups exceeded prevailing population norms [58].

The results may help inform other uncertainties of past research. In particular, immortal time bias can exaggerate life-expectancy estimates related to more years before rather than after an event; however, past analyses may not have considered individuals also need to survive long enough to be eligible for an event [59]. Multistate models can adjust for complex dynamics including time-dependent bias; however, these models need to further recognize that individuals might also require sustained earlier survival to be selected into a study [60]. Healthier people may have longer baselines regardless of status; however, analyses sometimes lack sufficient statistical power to check whether trends are significant [61, 62]. Our new analysis helps address these limitations in past survival statistics.

A limitation of our analysis is that statistics adjusting for immortal time can bias results in the opposite direction. Specifically, an individual does not always need to be alive to be nominated or win an Academy award (examples of posthumous recognition include Heath Ledger, James Dean, Chadwick Boseman, Peter Finch, Jeanne Eagels). The possible time lag can be significant due to delays in film distribution and release (examples include Larry Russel who won a posthumous award for music 18 years after his death). Statistics adjusting for immortal time, therefore, may lead to underestimating the survival associated with winning an Academy award. Similarly, missing data on deaths among controls is a further bias potentially leading our analysis to underestimate survival differences.

In summary, this study supports the theory that social factors may be important determinants of health at extremes of status and, therefore, might influence health for patients who have intermediate levels of success. The health effects might not be entirely due to occupation, education, or medical care. Instead, an explanation might include that successful people have more ideal lifestyles or can avoid some harmful stress [63]. The findings on longevity observed in our study, of course, do not mean people should take acting lessons to improve their health or awards should be dispensed by clinicians. Instead, the data suggest that major success might contribute to individuals behaving in ways that could potentially mitigate the wear-and-tear that can accumulate over years.

## Supporting information

S1 Appendix Technical appendix. The purpose of this appendix is to provide an §1) overview of the analysis, §2) specific additional results, §3) core Python codes for multistate modeling, and §4) SAS codes for the validation analysis.
(PDF)
