# Supplementary material for: Long-term mortality of academy award winning actors and actresses
Source: PLoS One. 2022 Apr 13;17(4):e0266563. doi: 10.1371/journal.pone.0266563 (PMC9007384; doi:10.1371/journal.pone.0266563)
Supplement: S1 Appendix — The purpose of this appendix is to provide an §1) overview of the analysis, §2) specific additional results, §3) core Python codes for multistate modeling, and §4) SAS codes for the validation analysis. (PDF) [file pone.0266563.s001.pdf]

Technical Appendix

OVERVIEW

The purpose of this appendix is to provide an §1) overview of the analysis, §2) specific additional results, §3) core Python codes for multistate modeling, and §4) SAS codes for the validation analysis. This material is intended as background technical information for readers interested in further background on our analyses on the longevity of Oscar winning actors. In addition, readers are referred to the GitHub resource for more details on multistate modeling (https://meter1.readthedocs.io/en/latest/installation.html).

The most complicated sections of the appendix involves the Python codes that involve 8 files that show details for a multistate transition model. Each file includes annotation where possible to embed explanations. In addition, the codes are also colorized to help stay organized. Red tends to denote data cleaning and organizational steps. Green tends to denote analytic steps and strategies. No other special colours are used. These documents represent earlier versions of the coding now available on GitHub.

§1) Overview of the Analysis

Multistate Transition Model for Testing Survival

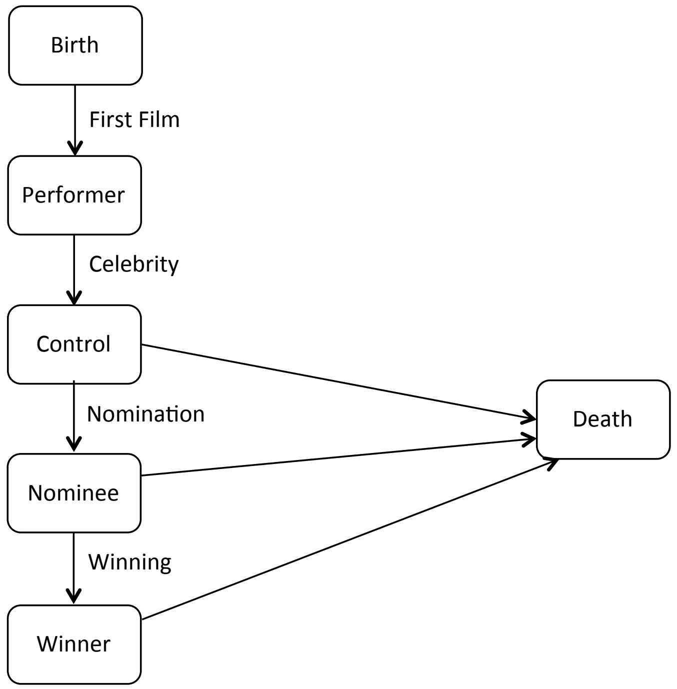

Figure 1. Footnote

Diagram of multistate transition model using rectangles to depict initial state (Birth), final state (Death), and four intermediate states (Performer, Control, Nominee, Winner). Transitions between states depicted as arrows. Life expectancy expressed in years and estimated from multistate life-table approach.

§2) Specific Additional Results

Table. Additional Predictors of Survival

CHARACTERISTIC		Life-expectancy for Group	Life-expectancy for Referrant	Difference	95% Confidence Interval
Social Status (referent is control)	Winner	81.3	76.2	5.1	3.3 to 7.3
Birth Era (referent is before 1920)	Recent	79.7	73.7	6.0	3.9 to 8.0
Sex (referent is male)	Female	78.3	75.1	3.2	1.0 to 5.5
Race (referent is white)	Non-white ¶	75.5	76.5	-1.0	-5.2 to 3.1
Birth Country (referent is USA)	Non-USA	78.4	76.0	2.4	0.0 to 4.5
Name (referent is unchanged)	Changed	78.8	76.3	2.5	0.6 to 5.7
Film Genre (referent is any other)	Drama	76.5	76.8	-0.3	-2.7 to 1.9

Footnote
* all estimates from Multi-state model
¶ includes asian, black, or other race

Figure: Analysis of Winners Compared to Nominees

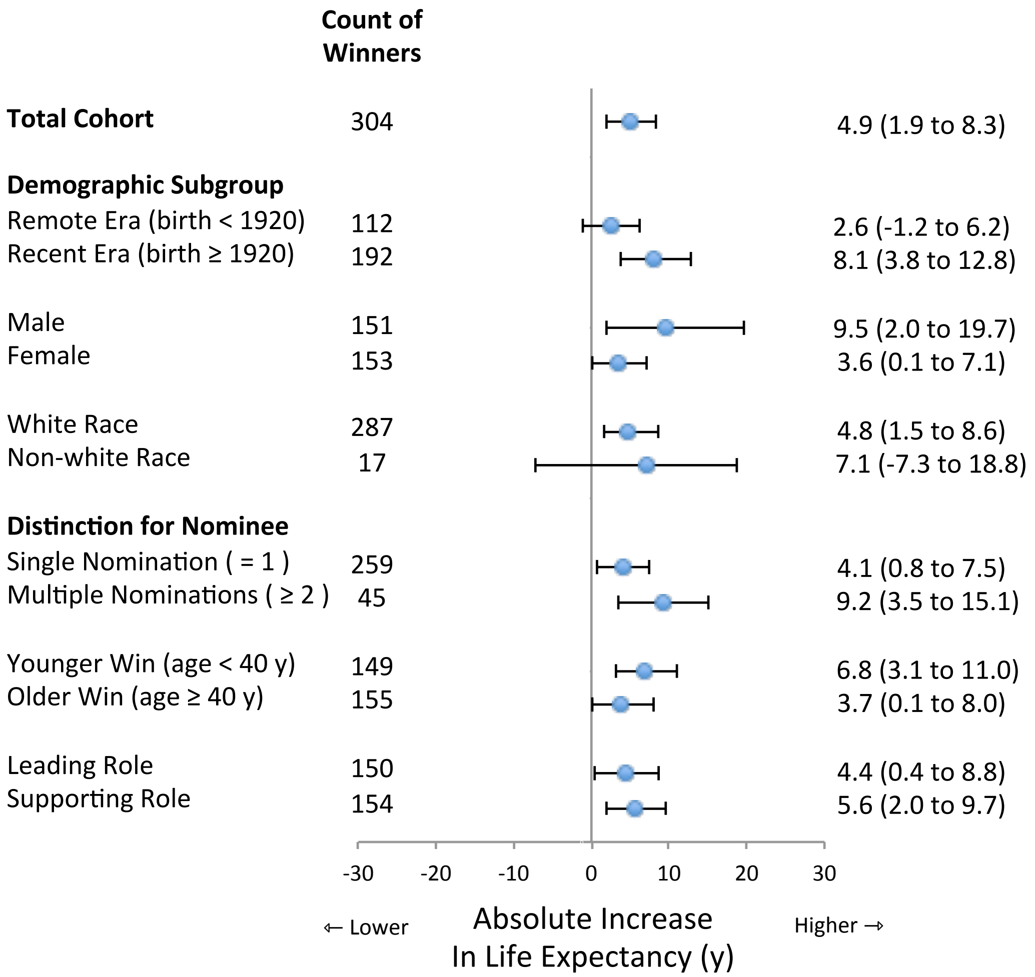

<u>Footnote</u>

Forest plot showing multiple sensitivity analyses of absolute increase in life-expectancy for winners in different subgroups. Referent group in all analyses is nominees and all controls excluded. Circles denote estimate and horizontal lines denote 95% confidence interval. Vertical line for null association marked as a 0 year increase in life expectancy. Sample size in each analysis shown as corresponding count of winners. Findings suggest increased life-expectancy for winners compared to nominees.

Figure: Analysis of Nominees compared to Controls

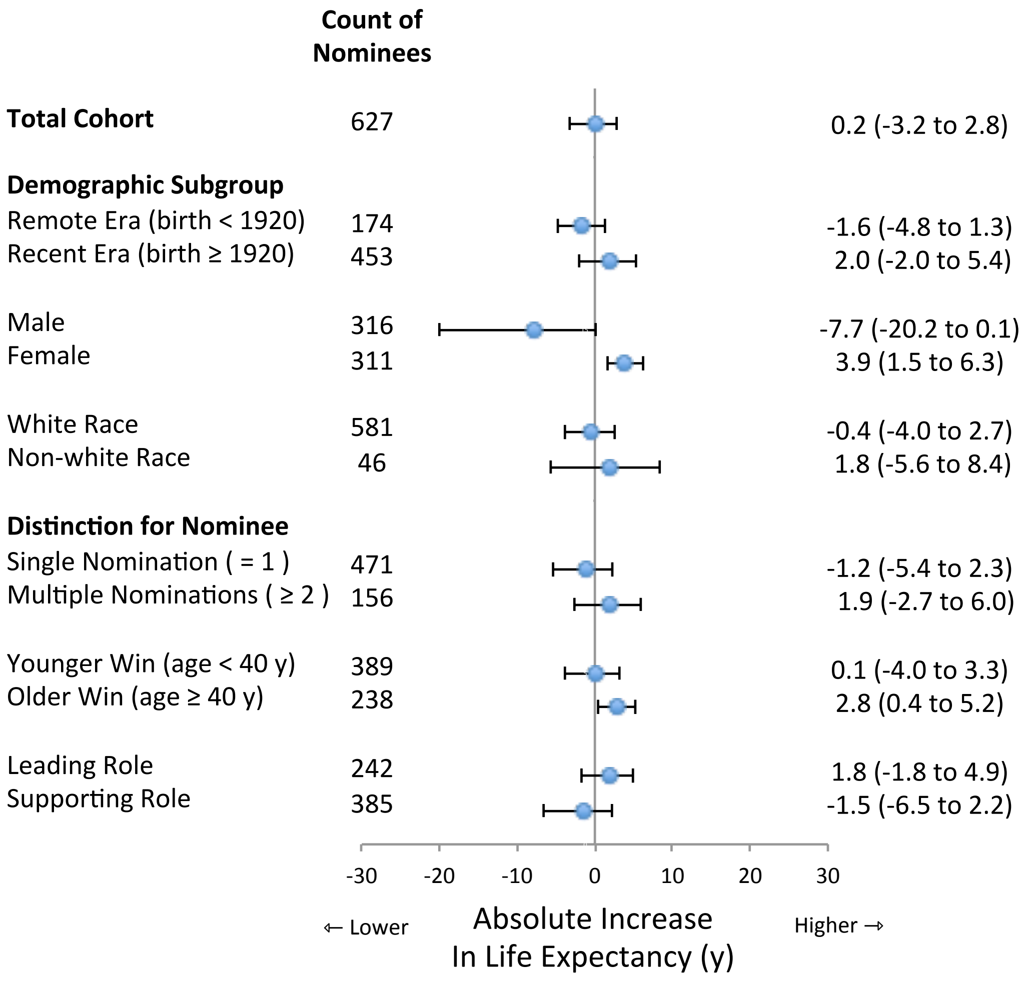

<u>Footnote</u>
Forest plot showing multiple sensitivity analyses of absolute increase in life-expectancy for nominees in different subgroups. Referent group in all analyses is control performers and all winners excluded. Circles denote estimate and horizontal lines denote 95% confidence interval. Vertical line for null association marked as a 0 year increase in life expectancy. Sample size in each analysis shown as corresponding count of winners. Findings suggest no significant increased life-expectancy for nominees compared to controls.

§3) Core Python Codes of Multistate Modeling

The first file is named PreliminaryDataCleanup and is intended to address performers who made more than one transition in a year (for example, first being nominated and first winning in the same year). In such cases, the person is classified by final state. An additional nuance is to computer each person's age at each transition based on the year of birth and year of transition. Keep in mind the coding shows the specific computer path and would need to be tailored for each programmer individually.

The second file is named MultiStateLifeTable and contains the core functions and subroutines. This name must not be changed since the details are called upon in later programs. The core features include creating the number of people at-risk at each transition (denominator) and the number of people making a transition at the corresponding age (numerator). This allows calculating a transition probability at each year (numerator / denominator). This also allows calculating a survivorship vector (membership of each group at each stage). Additional coding relates to transposing the data into a longitudinal format to allow calculation. Additional coding involves censoring individuals at corresponding stage (for example, transitioning from a nominee to a winner). Some final features involving exporting data for plotting and other analyses.

The third file is named WinnerVsNonWinnerMSM and contains much of the comparative analyses to contrast different groups. This name must not be changed because the details are called for later programs. The core features include using functions from the MultiStateLifeTable file to compare winners to non-winners. Incidentally, the group of non-winners can also be decomposed into the group of nominees and group of controls. This also introduces the bootstrap coding for calculating 95% confidence intervals around estimates. An additional set of codes is provided to compare conventional Kaplan-Meier analyses (no accounting for immortal time bias) to Multi-State analyses (accounting for immortal time bias).

The fourth file is named KaplaMeierDataOutput and contains the codes for creating the figure displaying survival over time in different groups. The core features begin by repairing 3 anomalous performers marked by posthumous nominations or posthumous wins (eg, Heath Ledger). Additional codes create survival probabilities at each year of life for controls, nominees, and winners. The functions used here are based on MultiStateLifeTable file.

The fifth file is named LifeExpectancyPredictors and contains the codes for examining additional predictors of survival (for example, sex and race). The main strategy is to calculate life-expectancy by subgroup analyses. The core subgroups are derived from the six baseline characteristics. Specifically, Birth Era (binary), Sex (binary), Race (binary), Country of Birth (binary), Name change (binary), and Drama film genre (binary). The general strategy remains unchanged based on calculating the number of performers at-risk (denominator) and number of transitions (numerator). The coding also again repairs the 3 anomalous performers marked by posthumous nominations or posthumous wins.

The sixth file is named StratifedAnalysesWithWinners and contains codes that enable testing the survival of winners compared to other performers. The core features include the Multi-State estimates of life-expectancies by concentrating on Winners and Controls (the group of Nominees is also included for completeness). The life-expectancy difference between Winners and Controls is calculated along with 95% confidence intervals. This difference is then subjected to secondary analyses based on stratification according to seven features. Specifically, Birth Era (binary), Sex (binary), Race (binary), Multiplicity (binary based on single win vs multiple wins), Age at first win (binary based age 40 years), and Role (binary based on lead vs supporting), and Other Recognitions (binary based on single nomination vs multiple nominations).

The seventh file is named StratifedAnalysesNoWinners and contains codes for testing the survival of nominees and control performers after excluding winners. In essence, performers were included until the year of nomination and were subsequently censored if they won at any time thereafter. These results are not presented in the main manuscript and are intended as an exploratory analysis to further examine the contrast between nominees and winners. The actual analyses and layout mirrors the StratifedAnalysesWithWinners file.

The final file is named SuperSubgroupAnalyses and contains codes for examining combination demographic subgroups distinguishing Birth era (binary), Sex (binary), Race (binary). This amounts to eight specific detailed stratified analyses (2 x 2 x 2). The core features rely on the MultiStateLifeTable file to evaluate the life-expectancy of winners, nominees, and controls in each group along with bootstrap simulation for all 95% confidence intervals. Each analysis

handles the demographic factor as a static characteristic and again estimates transition probabilities, a survival vector, and life-expectancy.

```
### This script takes the data in the original format (transitions for individual subjects recorded as calendar years)
### and adds additional rows to the dataset. For each performer we add 10 additional entries. For each of the 5 transitions
### excluding birth we record whether a performer ever made such a transition (recorded as 1 or 0) and if so, the age
### at which the transition was made. Located here is also our method for dealing with multiple transitions in a given
### year. If a performer makes more than 1 transition in a given year then the final transition supercedes. This is to
### say that if I transition from Performer -> Winner -> Death in one year then I am recorded as transitioning directly from
### nomination to death and my status as a winner is irrelevant.

import pandas as pd
import glob, os

path = r'C:/Users/NEVET/Desktop/Stars/python'
all = glob.glob(path + "/*.csv")
#d_frame = pd.DataFrame()

datafile = 'C:/Users/NEVET/Desktop/Stars/python/MoreForDeva2.csv'


df = pd.read_csv(datafile, names = ['Birth', 'First_Film', 'First_Ceremony', 'First_Nom', 'First_Win', 'Death', 'Final', 'Alive', 'Status',
'Male_1','Born_White','Born_USA','Name_Change','Drama_Genre','Lead_1','TWins','TNoms','MultiWinner','MultiNominee'], skiprows=1)

df.index.name = 'Locator'

df['final age'] = df['Final'] - df['Birth'] # creates a new column for the age at close-out of the study


states = {'First_Film': ['age of first Movie', 'first Movie status'], 'First_Ceremony': ['age of celebration', 'celebration status'], 'Death': ['age at death', 'death status'], 'First_Nom': ['age of nomination', 'nomination status'], 'First_Win': ['age of victory', 'victory status']}
# the above line simply tells the program what columns in the dataset that correspond to the new columns to be added

for ind in df.index: # this section ensures there is only one transition in a given year
    valid_lst = []
    value_lst = []
    for i in ['First_Film', 'First_Ceremony', 'First_Nom', 'First_Win']:
        if df[i][ind] is not None:
            valid_lst.append(i)
    for i in range(0, len(valid_lst) - 1): # if two transitions occur in one year, only the transition to the farther advanced state is kept
```

```
        for j in range(i+1,len(valid_lst)):
            if df[valid_lst[i]][ind] >= df[valid_lst[j]][ind]:
                df[valid_lst[i]][ind] = 10000
    for i in valid_lst:
        if df[i][ind] == 10000:
            df[i][ind] = None


for i in states: # this creates the new columns needed to stores the age-
based, as opposed to calendar year-based transition times
    df[states[i][0]] = ''
    df[states[i][1]] = ''


for ind in df.index:
    for i in states:
        if df[i][ind] != df[i][ind]: # if someone never reaches a state,
their time at reaching it is recorded as their final age to avoid NaNs,
but their status is recorded as negative
            df[states[i][0]][ind] = df['final age'][ind]
            df[states[i][1]][ind] = 0
        else:
            df[states[i][0]][ind] = df[i][ind] - df['Birth'][ind] # if
you do reach a state, we record your status as positive (1) and the age
at reaching that state is the year at reaching it minus birth year
            df[states[i][1]][ind] = 1



df.to_csv('C:/Users/NEVET/Desktop/Stars/python/OscarsDataMod.csv')
```

```
import numpy as np
import pandas as pd
import matplotlib.pyplot as plt
import copy
import string

### This script provides all of the basic functions necessary for Multi-
State Life Tables. The critical functions are:
### atrisk_and_transitions, transitionprobs_and_samplesizes,
survivorship_vector, and life_expectancy. atrisk_and_transitions
### records the risk sets at every given age, which corresponds to the
number of people in each of the states at every given age.
### It also records the number of performers who transition from any
given state to any other state at a given age.
### transitionprobs_and_samplesizes uses the output of this function to
compute the transition probabilities at every age
### and also records the associated sample sizes. survivorship_vector
takes a radix, a vector representing the initial
### distribution of individuals over states to make predictions for, and
an initial age, and outputs the expected
### evolution of that set of individuals over time.


def allowed_transitions(states):
    # INPUTS
    # "states":  a python list with the set of states in a given model
(eg. ["Person", "Performer", "Nominee", "Dead"])
    # this function takes a set of states and uses it to compute the
allowed transitions
    # it assumes the model is acyclic ie. individuals can only transition
towards states
    # to the right of it in the list
    lst = []
    for i in range(0, len(states)):
        for j in range(i+1, len(states)):
            lst.append([i, j])
    return lst


def df_to_list(data):
    # INPUTS
    # "data": a pandas dataframe (eg. the original data imported using
the pd.read_csv function)
    row_list = []  # want to convert data to python lists to speed up
computation
    for i in range((data.shape[0])):
        row_list.append(list(data.iloc[i, :]))
    return row_list


def atrisk_and_transitions(transition_ages, states, transition_statuses,
data, allowable_transitions, row_list):
    # A function to get the number of individuals in a given state at a
given age, and
```

```
    # number of transitions at each age.
    # INPUTS
    # "states": a python list with the set of states in a given model
(eg. ["Person", "Performer", "Nominee", "Dead"])
    # "transition_ages": a dictionary matching each state to a column in
the data set showing what age the performer reaches the state at
    # (eg. {"person": "age at birth", "performer": "age of first film",
"nominee": "age of nomination", "dead": "age at death"})
    # "transition_statuses": a dictionary matching each state to a column
in the data set showing whether a performer ever reached a state
    # {"person": 'birth status', "performer": "first film status",
"nominee": "age of nomination", "dead": "death status"}
    # "data": the data in expanded format with one performer per row -
this file is the output from datacleanup
    # "allowable_transitions":a list of allowable transitions in the
model with each element as a two-element list containing the
    # ordinal number of the states that make up the transition. eg.
[[0,1], [0,2], [0,3], [1,2], [1,3], [2,3]]
    # "row_list" should be the data in list format which can be generated
using df_to_list.
    column_positions = {}
    for i in range(0, len(data.columns)):
        column_positions[data.columns[i]] = i
    max_age = data['final age'].max() # need the final age so we know how
many age specific arrays needed

    at_risk = []
    transitions = []
    for i in range(0, max_age+1): # create an array containing the number
of people in each state for every age in the study
        at_risk.append([])
        transitions.append([[0 for x in range(len(states))] for y in
range(len(states))])
    for i in at_risk:
        for z in states:
            i.append(0)
    at_risk[0][0] = len(data) # initially, everyone is in state 1
    for i in row_list:
        current_state = 0
        perf_max_age = i[column_positions["final age"]]
        n = 1
        while n <= perf_max_age:
            at_risk[n][current_state] += 1
            for z in range(current_state, len(states)):
                if i[column_positions[transition_ages[states[z]]]] == n
and i[column_positions[transition_statuses[states[z]]]] == 1:
                    transitions[n][current_state][z] += 1
                    current_state = z
            n += 1
    riskdf = pd.DataFrame(at_risk, columns=states)
    riskdf.index.name = 'age'
    transitionsdf = []
    for z in range(0, max_age+1):
        translst = []
```

```
        for i in allowable_transitions:
            translst.append(transitions[z][i[0]][i[1]])
        transitionsdf.append(translst)
    names = []
    for i in allowable_transitions:
        names.append(states[i[0]] + "->" + states[i[1]])
    transdf = pd.DataFrame(transitionsdf, columns=names)
    transdf.index.name = 'age'
    return([riskdf, transdf])


def transitionprobs_and_samplesizes(atrisk, transitions,
allowable_transitions, states):
    # INPUTS
    # "atrisk": a list of the number of individuals in each state,
indexed at each age. This is the first output
    # from the at_risk_and_transitions function
    # "transitions": a list of the number of performers who made a given
transition at each age for each possible transition
    # this is the second output from the at_risk_and_transitions function
    # "allowable_transitions":a list of allowable transitions in the
model with each element as a two-element list containing the
    # ordinal number of the states that make up the transition. eg.
[[0,1], [0,2], [0,3], [1,2], [1,3], [2,3]]
    # "states": a python list with the set of states in a given model
(eg. ["Person", "Performer", "Nominee", "Dead"])
    transmat = [] # list for storing the time-dependent transition
probabilities
    samplesizes = [] # list for storing the sample size
    numstates = len(states)
    for ind in atrisk.index:
        transmat.append(np.full((numstates, numstates), 0, dtype=float))
        samplesizes.append(np.full((numstates, numstates), 0))
        for n in range(0, len(allowable_transitions)):
            fromstate = states[allowable_transitions[n][0]]
            tostate = states[allowable_transitions[n][1]]
            transition = fromstate + "->" + tostate
            if atrisk[fromstate][ind] > 0:
                samplesizes[ind][allowable_transitions[n][1],
allowable_transitions[n][0]] = atrisk[fromstate][ind]
                transmat[ind][allowable_transitions[n][1],
allowable_transitions[n][0]] =
np.true_divide(transitions[transition][ind], atrisk[fromstate][ind])
            else:
                samplesizes[ind][allowable_transitions[n][1],
allowable_transitions[n][0]] = atrisk[fromstate][ind]
                transmat[ind][allowable_transitions[n][1],
allowable_transitions[n][0]] = 0
    for i in range(0, numstates):
        transitions_out = []
        for z in allowable_transitions:
            if i == z[0]:
                transitions_out.append(z)
        for x in range(0, len(transmat)):
```

```
            stay_prob = 1
            for q in transitions_out:
                stay_prob -= transmat[x][q[1], q[0]]
            transmat[x][i,i] = stay_prob
    return [transmat, samplesizes]


def transitionsexport(transmat, samplesizes, states):
    # INPUTS
    # "transmat": a list of matrices that represent the transition
probabilities at each age. This is the first output from
    # transitionprobs_and_samplesizes
    # "samplesizes": a list of matrices with each of the sample sizes
corresponding to each of the probabilities. This is the
    # second output from transitionprobs_and_samplesizes
    # "states":  a python list with the set of states in a given model
(eg. ["Person", "Performer", "Nominee", "Dead"])
    allowable_transitions = allowed_transitions(states)
    for i in range(0, len(states)-1):
        rows = []
        column_names = []
        transitions_out = []
        for z in allowable_transitions:
            if i == z[0]:
                transitions_out.append(z)
                column_names.append(states[z[0]] + "->" + states[z[1]])
        for y in range(0, len(transmat)):
            row = []
            for x in transitions_out:
                row.append(transmat[y][x[1], x[0]])
            row.append(samplesizes[y][transitions_out[0][1],
transitions_out[0][0]])
            rows.append(row)
        column_names.append("sample size")
        statdf = pd.DataFrame(rows, columns=column_names)
        statdf.index.name = "age"
        statdf.to_csv ("C:/Users/NEVET/Desktop/Stars/python/" + states[i]
+ "_transitions.csv")


def survivorship_vector(transmat, radix, initial_age, states):
    # INPUTS
    # "transmat": a list of numpy matrices that represent the transition
probabilities at each age. This is the first output from
    #  transitionprobs_and_samplesizes
    # "radix": an initial condition for the number of performers in each
state we want to model, specified as a numpy vector.
    # For example, if we wanted the probabilities of being in each state
for 1 individual starting as a winner in the full model
    # the radix would be generated by np.array([[0],[0],[0],[0],[1],[0]],
dtype=float)
    # "initial_age": the initial age to model the survivorship outcomes
from, so an integer from 0 to 104 (oldest age in the dataset)
```

```
    # "states":  a python list with the set of states in a given model
(eg. ["Person", "Performer", "Nominee", "Dead"])
    rows = []
    l = radix
    for i in range(initial_age, len(transmat)):
        l = np.matmul(transmat[i], l)
        rows.append((l.transpose().flatten().tolist()))
    survdf = pd.DataFrame(rows, columns=states)
    survdf.index.name = "age"
    survdf.index += initial_age
    return survdf


def export_survivorship(survdf, name):
    # INPUTS
    # "survdf": a dataframe containing the given probabilities of being
in each state at each age for some initial condition.
    # this is precisely the output of survivorship_vector
    # "name": the name of the file (with extension) that you want to
output
    survdf.to_csv("C:/Users/NEVET/Desktop/Stars/python/" + name)


def survivorship_graph(survdf, states, order, colors):
    # INPUTS
    # "survdf": a dataframe containing the given probabilities of being
in each state at each age for some initial condition.
    # this is precisely the output of survivorship_vector
    # "states":  a python list with the set of states in a given model
(eg. ["Person", "Performer", "Nominee", "Dead"])
    # "order": the order you want the states to be from top to bottom in
the graph.
    # (eg. I find the most aesthetic option to be [5,4,3,2,1,0] for the
full model)
    # "colors": a list of colors accepted by matplotlib (as strings) that
matches the number of states
    survacc = survdf.divide(survdf.sum(axis=1), axis=0)
    for i in states:
        survacc[i] *= 100
    newstates = []
    for i in order:
        newstates.append(states[i])
    barWidth = 1.0
    plt.bar(survacc.index, survacc[newstates[0]], color=colors[0],
edgecolor=colors[0], width=barWidth, label=newstates[0])
    bot = survacc[newstates[0]]
    for i in range(1, len(newstates)):
        plt.bar(survacc.index, survacc[newstates[i]], bottom=bot,
color=colors[i], edgecolor=colors[i], width=barWidth, label=newstates[i])
        bot += survacc[newstates[i]]
    plt.legend(loc='upper left', bbox_to_anchor=(1, 1), ncol=1,
title="State")
    plt.box(on=False)
    plt.subplots_adjust(right=0.75)
```

```
    plt.margins(0, 0)
    plt.title("Probability of being in State by Age")
    plt.xlabel("Age, y")
    plt.ylabel("Probability, %")
    plt.show()


def censor_at_winner(data, data_as_list): # censoring every winner at the
age they won in order to construct a 4 state model for nominee life
expectancy
    # INPUTS
    # "data": the data in expanded format with one performer per row -
this file is the output from datacleanup
    # "data_as_list" should be the data in list format which can be
generated using df_to_list.
    column_positions = {}
    for i in range(0, len(data.columns)):
        column_positions[data.columns[i]] = i
    newdata = copy.deepcopy(data_as_list)
    for i in range(0, len(data_as_list)):
        if data_as_list[i][column_positions['victory status']] == 1:
            newdata[i][column_positions['final age']] =
newdata[i][column_positions['age of victory']]
            newdata[i][column_positions['death status']] = 0
            newdata[i][column_positions['age at death']] = float("NaN")
    return newdata


def censor_at_nominee(data, data_as_list):
    # INPUTS
    # "data": the data in expanded format with one performer per row -
this file is the output from datacleanup
    # "data_as_list" should be the data in list format which can be
generated using df_to_list.
    column_positions = {}
    for i in range(0, len(data.columns)):
        column_positions[data.columns[i]] = i
    newdata = copy.deepcopy(data_as_list)
    for i in range(0, len(data_as_list)):
        if data_as_list[i][column_positions['victory status']] == 1:
            newdata[i][column_positions['final age']] =
newdata[i][column_positions['age of victory']]
            newdata[i][column_positions['death status']] = 0
            newdata[i][column_positions['age at death']] = float("NaN")
        if data_as_list[i][column_positions['nomination status']] == 1:
            newdata[i][column_positions['final age']] =
newdata[i][column_positions['age of nomination']]
            newdata[i][column_positions['death status']] = 0
            newdata[i][column_positions['age at death']] = float("NaN")
    return newdata


def life_expectancy(transmat, state, age, states):
    # INPUTS
```

```
    # "transmat": a list of matrices that represent the transition
probabilities at each age. This is the first output from
    # transitionprobs_and_samplesizes
    # "age": the initial age to model the survivorship outcomes from, so
an integer from 0 to 104 (oldest age in the dataset)
    # note that if the age is not 0, the output life_ex corresponds to
the life expectancy from that point on not the total life expectancy
    # "state": the initial state to model the life expectancy from (eg.
"Performer")
    # "states": a python list with the set of states in a given model
(eg. ["Person", "Performer", "Nominee", "Dead"])
    radix = np.zeros((len(states), 1), dtype=float)
    radix[states.index(state), 0] = 1
    survdf = survivorship_vector(transmat, radix, age, states)
    surv = df_to_list(survdf)
    life_ex = 0
    for i in surv:
        life_ex += 1 - i[-1]
    return life_ex


def nomineewinnerKaplanMeier(data):
    # INPUTS
    # "data": the data in expanded format with one performer per row -
this file is the output from datacleanup
    curveNom = []
    s = 1
    for n in range(0, int(data[data['Status'] == 'Nominee']['final
age'].max() + 1)):
        curveNom.append(s)
        D = len(data[(data['age at death'] == n) & (data['Status'] ==
'Nominee') & (data['death status'] == 1)])
        N = len(data[(data['final age'] >= n - 1) & (data['Status'] ==
'Nominee')])
        s *= (N - D) / N
    curveWin = []
    s = 1
    for n in range(0, int(data[data['Status'] == 'Winner']['final
age'].max() + 1)):
        curveWin.append(s)
        D = len(data[(data['age at death'] == n) & (data['death status']
== 1) & (data['Status'] == 'Winner')])
        N = len(data[(data['final age'] >= n - 1) & (data['Status'] ==
'Winner')])
        s *= (N - D) / N
    curveControl = []
    s = 1
    for n in range(0, int(data[data['Status'] == 'Control']['final
age'].max() + 1)):
        curveControl.append(s)
        D = len(data[(data['age at death'] == n) & (data['death status']
== 1) & (data['Status'] == 'Control')])
        N = len(data[(data['final age'] >= n - 1) & (data['Status'] ==
'Control')])
```

```
        s *= (N - D) / N
    totalControl = 0
    totalNom = 0
    totalWin = 0
    for i in range(0, len(curveWin)):
        totalWin += curveWin[i]
    for i in range(0, len(curveNom)):
        totalNom += curveNom[i]
    for i in range(0, len(curveControl)):
        totalControl += curveControl[i]
    return [totalNom, totalWin, totalControl, curveNom, curveWin,
curveControl]


def nomineewinnerlifeexpectancy(data, transition_ages,
transition_statuses):
    # INPUTS
    # "data": the data in expanded format with one performer per row -
this file is the output from datacleanup
    # "transition_ages": a dictionary matching each state to a column in
the data set showing what age the performer reaches the state at
    # (eg. {"person": "age at birth", "performer": "age of first film",
"nominee": "age of nomination", "dead": "age at death"})
    # "transition_statuses": a dictionary matching each state to a column
in the data set showing whether a performer ever reached a state
    # {"person": 'birth status', "performer": "first film status",
"nominee": "age of nomination", "dead": "death status"}
    states = ['person', 'performer', 'celebrity', 'nominee', 'winner',
'dead']
    statesNom = ['person', 'performer', 'celebrity', 'nominee', 'dead']
    statesCon = ['person', 'performer', 'celebrity', 'dead']

    rowsFull = df_to_list(data)
    rowsToNom = censor_at_winner(data, rowsFull)
    rowsToCon = censor_at_nominee(data, rowsFull)

    allowable_transitions = allowed_transitions(states)
    allowable_transitionsNom = allowed_transitions(statesNom)
    allowable_transitionsCon = allowed_transitions(statesCon)

    risktransFull = atrisk_and_transitions(transition_ages, states,
transition_statuses, data, allowable_transitions, rowsFull)
    risktransToNom = atrisk_and_transitions(transition_ages, statesNom,
transition_statuses, data, allowable_transitionsNom, rowsToNom)
    risktransToCon = atrisk_and_transitions(transition_ages, statesCon,
transition_statuses, data, allowable_transitionsCon, rowsToCon)

    riskW = risktransFull[0]
    transW = risktransFull[1]

    riskN = risktransToNom[0]
    transN = risktransToNom[1]

    riskC  = risktransToCon[0]
```

```
    transC = risktransToCon[1]

    #riskW.to_csv("C:/Users/NEVET/Desktop/Stars/python/riskW.csv")
    #transW.to_csv("C:/Users/NEVET/Desktop/Stars/python/transW.csv")

    #riskN.to_csv("C:/Users/NEVET/Desktop/Stars/python/riskN.csv")
    #transN.to_csv("C:/Users/NEVET/Desktop/Stars/python/transN.csv")

    #riskC.to_csv("C:/Users/NEVET/Desktop/Stars/python/riskC.csv")
    #transC.to_csv("C:/Users/NEVET/Desktop/Stars/python/transC.csv")

    probsFull = transitionprobs_and_samplesizes(risktransFull[0],
risktransFull[1], allowable_transitions, states)
    probsToNom = transitionprobs_and_samplesizes(risktransToNom[0],
risktransToNom[1], allowable_transitionsNom, statesNom)
    probsToCon = transitionprobs_and_samplesizes(risktransToCon[0],
risktransToCon[1], allowable_transitionsCon, statesCon)

    radixFull = np.array([[0],[0],[0],[0],[1],[0]], dtype=float)
    radixToNom = np.array([[0],[0],[0],[1],[0]], dtype=float)
    radixToCon = np.array([[0], [0], [1], [0]], dtype=float)

    survFull = survivorship_vector(probsFull[0], radixFull, 0, states)
    survToNom = survivorship_vector(probsToNom[0], radixToNom, 0,
statesNom)
    survToCon = survivorship_vector(probsToCon[0], radixToCon, 0,
statesCon)

    #survFull.to_csv("C:/Users/NEVET/Desktop/Stars/python/survW.csv")
    #survToNom.to_csv("C:/Users/NEVET/Desktop/Stars/python/survN.csv")
    #survToCon.to_csv("C:/Users/NEVET/Desktop/Stars/python/survC.csv")


    nomLifeEx = life_expectancy(probsToNom[0], 'nominee', 0, statesNom)
    winLifeEx = life_expectancy(probsFull[0], 'winner', 0, states)
    conLifeEx = life_expectancy(probsToCon[0], 'celebrity', 0, statesCon)
    return [nomLifeEx, winLifeEx, conLifeEx]


def KMvsMultiState(data, transition_ages, transition_statuses):
    # INPUTS
    # "data": the data in expanded format with one performer per row -
this file is the output from datacleanup
    # "transition_ages": a dictionary matching each state to a column in
the data set showing what age the performer reaches the state at
    # (eg. {"person": "age at birth", "performer": "age of first film",
"nominee": "age of nomination", "dead": "age at death"})
    # "transition_statuses": a dictionary matching each state to a column
in the data set showing whether a performer ever reached a state
    # {"person": 'birth status', "performer": "first film status",
"nominee": "age of nomination", "dead": "death status"}
    rowsFull = df_to_list(data)
    rowsToNom = censor_at_winner(data, rowsFull)
    rowsToCon = censor_at_nominee(data, rowsFull)
```

```
    states = ['person', 'performer', 'celebrity', 'nominee', 'winner',
'dead']
    statesNom = ['person', 'performer', 'celebrity', 'nominee', 'dead']
    statesCon = ['person', 'performer', 'celebrity', 'dead']
    allowable_transitions = allowed_transitions(states)
    allowable_transitionsNom = allowed_transitions(statesNom)
    allowable_transitionsCon = allowed_transitions(statesCon)

    risktransFull = atrisk_and_transitions(transition_ages, states,
transition_statuses, data, allowable_transitions, rowsFull)
    risktransToNom = atrisk_and_transitions(transition_ages, statesNom,
transition_statuses, data, allowable_transitionsNom, rowsToNom)
    risktransToCon = atrisk_and_transitions(transition_ages, statesCon,
transition_statuses, data, allowable_transitionsCon, rowsToCon)


    probsFull  = transitionprobs_and_samplesizes(risktransFull[0],
risktransFull[1], allowable_transitions, states)
    probsToNom = transitionprobs_and_samplesizes(risktransToNom[0],
risktransToNom[1], allowable_transitionsNom, statesNom)
    probsToCon = transitionprobs_and_samplesizes(risktransToCon[0],
risktransToCon[1], allowable_transitionsCon, statesCon)

    radixFull = np.array([[0],[0],[0],[0],[1],[0]], dtype=float)
    radixToNom = np.array([[0],[0],[0],[1],[0]], dtype=float)
    radixToCon = np.array([[0], [0], [1], [0]], dtype=float)

    survFull = survivorship_vector(probsFull[0], radixFull, 0, states)
    survToNom = survivorship_vector(probsToNom[0], radixToNom, 0,
statesNom)
    survToCon = survivorship_vector(probsToCon[0], radixToCon, 0,
statesCon)


    KM = nomineewinnerKaplanMeier(data)
    survFull['alive'] = 1 - survFull['dead']
    survToNom['alive'] = 1 - survToNom['dead']
    survToCon['alive'] = 1 - survToCon['dead']
    nomKM = KM[3]
    winKM = KM[4]
    conKM = KM[5]
    nomMSM = survToNom['alive'].tolist()
    winMSM = survFull['alive'].tolist()
    conMSM = survToCon['alive'].tolist()
    plt.step(range(0,len(nomKM)), nomKM, marker='', color='red',
linewidth=1, linestyle='dashed', label='Nominee Survival K-M')
    plt.step(range(0,len(nomMSM)), nomMSM, marker='', color='red',
linewidth=1, label='Nominee Survival Multi-State')
    plt.legend()
    plt.title("Nominee Survival, Kaplan-Meier vs Multi-State Model")
    plt.xlabel("Age, y")
    plt.ylabel("Proportion Surviving")
    plt.show()
```

```
    plt.step(range(0, len(winKM)), winKM, marker='', color='red',
linewidth=1, linestyle='dashed', label='Winner Survival K-M')
    plt.step(range(0, len(winMSM)), winMSM, marker='', color='red',
linewidth=1, label='Winner Survival Multi-State')
    plt.legend()
    plt.title("Winner Survival, Kaplan-Meier vs Multi-State Model")
    plt.xlabel("Age, y")
    plt.ylabel("Proportion Surviving")
    plt.show()
    plt.step(range(0, len(nomMSM)), nomMSM, marker='', color='blue',
linewidth=1,label='Nominee Survival')
    plt.step(range(0, len(winMSM)), winMSM, marker='', color='red',
linewidth=1, label='Winner Survival')
    plt.legend()
    plt.title("Nominee vs Winner Survival, Multi-State Model")
    plt.xlabel("Age, y")
    plt.ylabel("Proportion Surviving")
    plt.show()
    plt.step(range(0, len(nomKM)), nomKM, marker='', color='blue',
linewidth=1, label='Nominee Survival')
    plt.step(range(0, len(winKM)), winKM, marker='', color='red',
linewidth=1, label='Winner Survival')
    plt.legend()
    plt.title("Nominee vs Winner Survival, Kaplan-Meier Model")
    plt.xlabel("Age, y")
    plt.ylabel("Proportion Surviving")
    plt.show()


def bootstrap_life_ex(data, transition_ages, transition_statuses, n,
outfile):
    # INPUTS
    # "data": the data in expanded format with one performer per row -
this file is the output from datacleanup
    # "transition_ages": a dictionary matching each state to a column in
the data set showing what age the performer reaches the state at
    # (eg. {"person": "age at birth", "performer": "age of first film",
"nominee": "age of nomination", "dead": "age at death"})
    # "transition_statuses": a dictionary matching each state to a column
in the data set showing whether a performer ever reached a state
    # {"person": 'birth status', "performer": "first film status",
"nominee": "age of nomination", "dead": "death status"}
    # "n": the number of bootstraps to run
    # "outfile": the name of the file to output the bootstrap results to
(extension included)
    rows = []
    KMresult = nomineewinnerKaplanMeier(data)

    NomineeKM = KMresult[3]
    WinnerKM  = KMresult[4]
    ControlKM = KMresult[5]

    NomineeKMdf = pd.DataFrame(NomineeKM, columns=['NomineeKM'])
    WinnerKMdf  = pd.DataFrame(WinnerKM,  columns=['WinnerKM'])
```

```
    ControlKMdf = pd.DataFrame(ControlKM, columns=['ControlKM'])

    #NomineeKMdf.to_csv("C:/Users/NEVET/Desktop/Stars/python/" + data +
"NomineeKM.csv")
    #WinnerKMdf.to_csv("C:/Users/NEVET/Desktop/Stars/python/" + data +
"WinnerKM.csv")
    #ControlKMdf.to_csv("C:/Users/NEVET/Desktop/Stars/python/" + data +
"ControlKM.csv")


#NomineeKMdf.to_csv("C:/Users/NEVET/Desktop/Stars/python/NomineeKM.csv")

#WinnerKMdf.to_csv("C:/Users/NEVET/Desktop/Stars/python/WinnerKM.csv")

#ControlKMdf.to_csv("C:/Users/NEVET/Desktop/Stars/python/ControlKM.csv")


    result = nomineewinnerlifeexpectancy(data, transition_ages,
transition_statuses)
    rows.append(result+[KMresult[0],KMresult[1], KMresult[2]])

    for i in range(0, len(rows[0])):
        rows[0][i] = round(rows[0][i], 1)
    print("BEST ESTIMATE")
    print("Nominee Life Expectancy: " + str(result[0]))
    print("Winner Life Expectancy: " + str(result[1]))
    print("Control Life Expectancy: "+ str(result[2]))
    print("Nominee Simple KM: " + str(KMresult[0]))
    print("Winner Simple KM: " + str(KMresult[1]))
    print("Control Simple KM: " + str(KMresult[2]))

    for i in range(0, n):
        boot_df = data.sample(frac = 1, replace = True)
        result = nomineewinnerlifeexpectancy(boot_df, transition_ages,
transition_statuses)
        KMresult = nomineewinnerKaplanMeier(boot_df)
        rows.append(result+[KMresult[0],KMresult[1], KMresult[2]])
        print("BOOT RUN " + str(i+1))
        print("Nominee Life Expectancy: " + str(result[0]))
        print("Winner Life Expectancy: " + str(result[1]))
        print("Control Life Expectancy: " + str(result[2]))
        print("Nominee Simple KM: " + str(KMresult[0]))
        print("Winner Simple KM: " + str(KMresult[1]))
        print("Control Simple KM: " + str(KMresult[2]))
    boot_results = pd.DataFrame(rows, columns=['Nominee Life Expectancy',
'Winner Life Expectancy', 'Control Life Expectancy', 'Nominee Simple KM',
'Winner Simple KM', 'Control Simple KM'])
    boot_results.index.name = "Bootstrap Run"
    boot_results.rename(index={0: 'Best Estimate'}, inplace=True)
    boot_results.to_csv(outfile)


def confidence_interval(data, column_name, confidence_level):
    # INPUTS
```

```
    # "data": the bootstrap results imported as a dataframe (eg. using
pd.read_csv). precisely the output file from bootstrap_life_ex
    # "column_name": the statistic that you want the interval for,
specified by the name of the column containing it (eg. "Nominee Life
Expectancy")
    # "confidence_level": a real number between 0 and 1 that represents
the desired confidence level. eg. 0.95 for 95%
    results = data[column_name].tolist()
    results.sort()
    lower_bound = int((1 - confidence_level) / 2 * len(results)) - 1
    upper_bound = int((confidence_level + 1) / 2 * len(results)) - 1
    if lower_bound < 0:
        lower_bound = 0
    return [round(float(results[lower_bound]), 1),
round(float(results[upper_bound]), 1)]


def summary_results(bootstrap, outfile):
    # INPUTS
    # "bootstrap": the bootstrap results imported as a dataframe (eg.
using pd.read_csv). precisely the output file from bootstrap_life_ex
    # "outfile": the name of the file to output the summary of the
bootstrap results to (extension included)
    nom_best = float(bootstrap['Nominee Life Expectancy']['Best
Estimate'])
    win_best = float(bootstrap['Winner Life Expectancy']['Best
Estimate'])
    con_best = float(bootstrap['Control Life Expectancy']['Best
Estimate'])
    nomKM_best = float(bootstrap['Nominee Simple KM']['Best Estimate'])
    winKM_best = float(bootstrap['Winner Simple KM']['Best Estimate'])
    conKM_best = float(bootstrap['Control Simple KM']['Best Estimate'])
    nomwin_best = round(win_best - nom_best, 1)
    wincon_best = round(win_best - con_best, 1)
    nomcon_best = round(nom_best - con_best, 1)
    nomwinKM_best = round(winKM_best - nomKM_best, 1)
    winconKM_best = round(winKM_best - conKM_best, 1)
    nomconKM_best = round(nomKM_best - conKM_best, 1)
    boot_df = bootstrap.drop(['Best Estimate', 'Bootstrap Run'])
    boot_df.index.name = 'Bootstrap Run'
    nom_msm = confidence_interval(boot_df, 'Nominee Life Expectancy',
0.95)
    win_msm = confidence_interval(boot_df, 'Winner Life Expectancy',
0.95)
    con_msm = confidence_interval(boot_df, 'Control Life Expectancy',
0.95)
    nom_km = confidence_interval(boot_df, 'Nominee Simple KM', 0.95)
    win_km = confidence_interval(boot_df, 'Winner Simple KM', 0.95)
    con_km = confidence_interval(boot_df, 'Control Simple KM', 0.95)
    boot_df['Nominee Winner Difference'] = pd.to_numeric(boot_df['Winner
Life Expectancy'], errors='ignore') - pd.to_numeric(boot_df['Nominee Life
Expectancy'], errors='ignore')
```

```
    boot_df['Control Winner Difference'] = pd.to_numeric(boot_df['Winner
Life Expectancy'], errors='ignore') - pd.to_numeric(boot_df['Control Life
Expectancy'], errors='ignore')
    boot_df['Nominee Control Difference'] =
pd.to_numeric(boot_df['Nominee Life Expectancy'], errors='ignore') -
pd.to_numeric(boot_df['Control Life Expectancy'], errors='ignore')
    boot_df['Nominee Winner Difference KM'] =
pd.to_numeric(boot_df['Winner Simple KM'],errors='ignore') -
pd.to_numeric(boot_df['Nominee Simple KM'], errors='ignore')
    boot_df['Control Winner Difference KM'] =
pd.to_numeric(boot_df['Winner Simple KM'],errors='ignore') -
pd.to_numeric(boot_df['Control Simple KM'], errors='ignore')
    boot_df['Nominee Control Difference KM'] =
pd.to_numeric(boot_df['Nominee Simple KM'],errors='ignore') -
pd.to_numeric(boot_df['Control Simple KM'], errors='ignore')
    nomwindiff = confidence_interval(boot_df, 'Nominee Winner
Difference', 0.95)
    conwindiff = confidence_interval(boot_df, 'Control Winner
Difference', 0.95)
    nomcondiff = confidence_interval(boot_df, 'Nominee Control
Difference', 0.95)
    nomwinkm = confidence_interval(boot_df, 'Nominee Winner Difference
KM', 0.95)
    conwinkm = confidence_interval(boot_df, 'Control Winner Difference
KM', 0.95)
    nomconkm = confidence_interval(boot_df, 'Nominee Control Difference
KM', 0.95)
    MSMPointEstimates = [win_best, nom_best, con_best, nomwin_best,
wincon_best, nomcon_best]
    MSMConfIntervals = [win_msm, nom_msm, con_msm, nomwindiff,
conwindiff, nomcondiff]
    MSM_column = []
    for i in range(0, len(MSMPointEstimates)):
        MSM_column.append(str(MSMPointEstimates[i]) + " " +
str(MSMConfIntervals[i]))
    KMPointEstimates = [winKM_best, nomKM_best, conKM_best,
nomwinKM_best, winconKM_best, nomconKM_best]
    KMConfIntervals = [win_km, nom_km, con_km, nomwinkm, conwinkm,
nomconkm]
    KM_column = []
    for i in range(0, len(KMPointEstimates)):
        KM_column.append(str(KMPointEstimates[i]) + " " +
str(KMConfIntervals[i]))
    index = ['Winner Life Expectancy', 'Nominee Life Expectancy',
'Control Life Expectancy','Winner-Nominee Difference', 'Winner-Control
Difference', 'Nominee-Control Difference']
    sum_df = pd.DataFrame(list(zip(MSM_column, KM_column,
index)),columns=['Multi-State Modelling Method (Estimate [CI])', 'Kaplan
Meier Method (Estimate [CI])', 'Measure'])
    sum_df.set_index('Measure', inplace=True)
    sum_df.to_csv(outfile)
```

```
import MultiStateLifeTable as MSLT
import numpy as np
import pandas as pd
import matplotlib.pyplot as plt
import copy
import string


### This script, using many of the basic functions from
MultiStateLifeTable, runs the analysis for winners vs non-winners.
### Essentially, it uses two versions of the dataset: the full dataset
and the dataset with winners censored at date of award.
### In order to run the analysis in stages, unlike MultiStateLifeTable,
it has functionality for specifying which specific
### states to use in the model (as an input to the functions
bootstrap_life_ex2 and KMvsMS). It also contains a basic function
### for computing allowable transitions in a given model based on which
states are included.

def WinnerNonWinnerKaplanMeier(data):
    curveNon = []
    s = 1
    for n in range(0, int(data[data['Status'] != 'Winner']['final
age'].max() + 1)):
        curveNon.append(s)
        D = len(data[(data['age at death'] == n) & (data['Status'] !=
'Winner') & (data['death status'] == 1)])
        N = len(data[(data['final age'] >= n - 1) & (data['Status'] !=
'Winner')])
        s *= (N - D) / N
    curveWin = []
    s = 1
    for n in range(0, int(data[data['Status'] == 'Winner']['final
age'].max() + 1)):
        curveWin.append(s)
        D = len(data[(data['age at death'] == n) & (data['death status']
== 1) & (data['Status'] == 'Winner')])
        N = len(data[(data['final age'] >= n - 1) & (data['Status'] ==
'Winner')])
        s *= (N - D) / N
    totalNon = 0
    totalWin = 0
    for i in range(0, len(curveWin)):
        totalWin += curveWin[i]
    for i in range(0, len(curveNon)):
        totalNon += curveNon[i]
    return [totalNon, totalWin, curveNon, curveWin]


import pickle


def using_multiindex(A, columns):
    shape = A.shape
```

```
    index = pd.MultiIndex.from_product([range(s)for s in shape],
names=columns)
    dff = pd.DataFrame({'A': A.flatten()}, index=index).reset_index()
    return dff


def KMvsMS(data, states, transition_ages, transition_statuses,
comparison):

    rowsFull = MSLT.df_to_list(data)
    rowsToNom = MSLT.censor_at_winner(data, rowsFull)

    transitions_full = MSLT.allowed_transitions(states)

    statesToNom = copy.copy(states)
    del statesToNom[-2]

    transitions_nom = MSLT.allowed_transitions(statesToNom)

    risktransFull = MSLT.atrisk_and_transitions(transition_ages, states,
transition_statuses, data, transitions_full, rowsFull)
    risktransToNom = MSLT.atrisk_and_transitions(transition_ages,
statesToNom, transition_statuses, data, transitions_nom, rowsToNom)

    probsFull = MSLT.transitionprobs_and_samplesizes(risktransFull[0],
risktransFull[1], transitions_full,states)
    probsToNom = MSLT.transitionprobs_and_samplesizes(risktransToNom[0],
risktransToNom[1], transitions_nom,statesToNom)

    radixFull = np.zeros((len(states), 1), dtype=float)
    radixFull[states.index("winner"), 0] = 1
    radixNon = np.zeros((len(states) - 1, 1), dtype=float)
    radixNon[states.index(comparison), 0] = 1

    survFull = MSLT.survivorship_vector(probsFull[0], radixFull, 0,
states)
    survNon = MSLT.survivorship_vector(probsToNom[0], radixNon, 0,
statesToNom)

    KM = WinnerNonWinnerKaplanMeier(data)
    survFull['alive'] = 1 - survFull['dead']
    survNon['alive'] = 1 - survNon['dead']

    PersonKM = KM[2]
    winKM = KM[3]

    PersonMSM = survNon['alive'].tolist()
    winMSM = survFull['alive'].tolist()



    riskW2 = risktransFull[0]
    transW2 = risktransFull[1]
```

```
    riskN2 = risktransToNom[0]
    transN2 = risktransToNom[1]

    #riskW2.to_csv("C:/Users/NEVET/Desktop/Stars/python/riskW2a.csv")
    #transW2.to_csv("C:/Users/NEVET/Desktop/Stars/python/transW2a.csv")

    #riskN2.to_csv("C:/Users/NEVET/Desktop/Stars/python/riskN2a.csv")
    #transN2.to_csv("C:/Users/NEVET/Desktop/Stars/python/transN2a.csv")

    #survFull.to_csv("C:/Users/NEVET/Desktop/Stars/python/survW2a.csv")
    #survNon.to_csv("C:/Users/NEVET/Desktop/Stars/python/survN2a.csv")


    plt.step(range(0, len(PersonKM)), PersonKM, marker='', color='red',
linewidth=1, linestyle='dashed',
             label='Non-Winner Survival K-M')
    plt.step(range(0, len(PersonMSM)), PersonMSM, marker='', color='red',
linewidth=1, label='Non-Winner Survival Multi-State')
    plt.legend()
    plt.title("Non-Winner Survival, Kaplan-Meier vs Multi-State Model")
    plt.xlabel("Age, y")
    plt.ylabel("Proportion Surviving")
    plt.show()
    plt.step(range(0, len(winKM)), winKM, marker='', color='red',
linewidth=1, linestyle='dashed',
             label='Winner Survival K-M')
    plt.step(range(0, len(winMSM)), winMSM, marker='', color='red',
linewidth=1, label='Winner Survival Multi-State')
    plt.legend()
    plt.title("Winner Survival, Kaplan-Meier vs Multi-State Model")
    plt.xlabel("Age, y")
    plt.ylabel("Proportion Surviving")
    plt.show()
    plt.step(range(0, len(PersonMSM)), PersonMSM, marker='',
color='blue', linewidth=1, label='Non-Winner Survival')
    plt.step(range(0, len(winMSM)), winMSM, marker='', color='red',
linewidth=1, label='Winner Survival')
    plt.legend()
    plt.title("Non-Winner vs Winner Survival, Multi-State Model")
    plt.xlabel("Age, y")
    plt.ylabel("Proportion Surviving")
    plt.show()
    plt.step(range(0, len(PersonKM)), PersonKM, marker='', color='blue',
linewidth=1, label='Non-Winner Survival')
    plt.step(range(0, len(winKM)), winKM, marker='', color='red',
linewidth=1, label='Winner Survival')
    plt.legend()
    plt.title("Non-Winner vs Winner Survival, Kaplan-Meier Model")
    plt.xlabel("Age, y")
    plt.ylabel("Proportion Surviving")
    plt.show()
```

```
def WinnerNonWinnerlifeexpectancy(data, states, transition_ages,
transition_statuses, comparison):
    rowsFull = MSLT.df_to_list(data)
    rowsToNom = MSLT.censor_at_winner(data, rowsFull)
    transitions_full = MSLT.allowed_transitions(states)
    statesToNom = copy.copy(states)
    del statesToNom[-2]
    transitions_nom = MSLT.allowed_transitions(statesToNom)
    risktransFull = MSLT.atrisk_and_transitions(transition_ages, states,
transition_statuses, data, transitions_full, rowsFull)
    risktransToNom = MSLT.atrisk_and_transitions(transition_ages,
statesToNom, transition_statuses, data, transitions_nom, rowsToNom)
    probsFull = MSLT.transitionprobs_and_samplesizes(risktransFull[0],
risktransFull[1], transitions_full, states)
    probsToNom = MSLT.transitionprobs_and_samplesizes(risktransToNom[0],
risktransToNom[1], transitions_nom, statesToNom)
    radixFull = np.array([[1],[0],[0],[0],[0],[0]], dtype=float)
    radixToNom = np.array([[1],[0],[0],[0],[0]], dtype=float)

    survFull = MSLT.survivorship_vector(probsFull[0], radixFull, 0,
states)
    survNon = MSLT.survivorship_vector(probsToNom[0], radixToNom, 0,
statesToNom)

    survFull['alive'] = 1 - survFull['dead']
    survNon['alive'] = 1 - survNon['dead']

    PersonMSM = survNon['alive'].tolist()
    winMSM = survFull['alive'].tolist()

    riskW2 = risktransFull[0]
    transW2 = risktransFull[1]

    riskN2 = risktransToNom[0]
    transN2 = risktransToNom[1]

    #riskW2.to_csv("C:/Users/NEVET/Desktop/Stars/python/riskW2b.csv")
    #transW2.to_csv("C:/Users/NEVET/Desktop/Stars/python/transW2b.csv")

    #riskN2.to_csv("C:/Users/NEVET/Desktop/Stars/python/riskN2b.csv")
    #transN2.to_csv("C:/Users/NEVET/Desktop/Stars/python/transN2b.csv")

    #survFull.to_csv("C:/Users/NEVET/Desktop/Stars/python/survW2b.csv")
    #survNon.to_csv("C:/Users/NEVET/Desktop/Stars/python/survN2b.csv")

    #MSLT.survivorship_graph(survFull, states, [5,4,3,2,1,0], ['black',
'gold', 'purple', 'darkgreen', 'green', 'limegreen'])
    #MSLT.survivorship_graph(survNon, statesToNom, [4,3,2,1,0], ['black',
'purple', 'darkgreen', 'green', 'limegreen'])
    nonLifeEx = MSLT.life_expectancy(probsToNom[0], comparison, 0,
statesToNom)
    winLifeEx = MSLT.life_expectancy(probsFull[0], 'winner', 0, states)
    return [nonLifeEx, winLifeEx]
```

```
def bootstrap_life_ex2(data, n, states, transition_ages, 
transition_statuses, comparison, outfile):
    rows = []

    KMresult = WinnerNonWinnerKaplanMeier(data)

    personKM = KMresult[2]
    winKM = KMresult[3]

    personKMdf = pd.DataFrame(personKM, columns=['PersonKM'])
    winKMdf    = pd.DataFrame(winKM, columns=['WinKM'])


#personKMdf.to_csv("C:/Users/NEVET/Desktop/Stars/python/person2KM.csv", 
index='Age')
    #winKMdf.to_csv("C:/Users/NEVET/Desktop/Stars/python/win2KM.csv", 
index='Age')

    result = WinnerNonWinnerlifeexpectancy(data, states, transition_ages, 
transition_statuses, comparison)

    rows.append(result+[KMresult[0],KMresult[1]])
    for i in range(0, len(rows[0])):
        rows[0][i] = round(rows[0][i], 1)
    print("BEST ESTIMATE")
    print("Non-Winner Life Expectancy: " + str(result[0]))
    print("Winner Life Expectancy: " + str(result[1]))
    print("Non-Winner Simple KM: " + str(KMresult[0]))
    print("Winner Simple KM: " + str(KMresult[1]))

    for i in range(0, n):
        boot_df = data.sample(frac = 1, replace = True)
        result = WinnerNonWinnerlifeexpectancy(boot_df, states, 
transition_ages, transition_statuses, comparison)
        KMresult = WinnerNonWinnerKaplanMeier(boot_df)
        rows.append(result+[KMresult[0],KMresult[1]])
        print("BOOT RUN " + str(i+1))
        print("Non-Winner Life Expectancy: " + str(result[0]))
        print("Winner Life Expectancy: " + str(result[1]))
        print("Non-Winner Simple KM: " + str(KMresult[0]))
        print("Winner Simple KM: " + str(KMresult[1]))
    boot_results = pd.DataFrame(rows, columns=['Non-Winner Life 
Expectancy', 'Winner Life Expectancy', 'Non-Winner Simple KM', 'Winner 
Simple KM'])
    boot_results.index.name = "Bootstrap Run"
    boot_results.rename(index={0: 'Best Estimate'}, inplace=True)
    boot_results.to_csv("C:/Users/NEVET/Desktop/Stars/python/" + outfile)


def summary_results(bootstrap, outfile):
    non_best = float(bootstrap['Non-Winner Life Expectancy']['Best 
Estimate'])
```

```
    win_best = float(bootstrap['Winner Life Expectancy']['Best
Estimate'])
    nonKM_best = float(bootstrap['Non-Winner Simple KM']['Best
Estimate'])
    winKM_best = float(bootstrap['Winner Simple KM']['Best Estimate'])
    winnon_best = round(win_best - non_best, 1)
    winnonKM_best = round(winKM_best - nonKM_best, 1)
    boot_df = bootstrap.drop(['Best Estimate', 'Bootstrap Run'])
    boot_df.index.name = 'Bootstrap Run'
    non_msm = MSLT.confidence_interval(boot_df, 'Non-Winner Life
Expectancy', 0.95)
    win_msm = MSLT.confidence_interval(boot_df, 'Winner Life Expectancy',
0.95)
    non_km = MSLT.confidence_interval(boot_df, 'Non-Winner Simple KM',
0.95)
    win_km = MSLT.confidence_interval(boot_df, 'Winner Simple KM', 0.95)
    boot_df['Non-Winner Winner Difference'] =
pd.to_numeric(boot_df['Winner Life Expectancy'], errors='ignore') -
pd.to_numeric(boot_df['Non-Winner Life Expectancy'], errors='ignore')
    boot_df['Non-Winner Winner Difference KM'] =
pd.to_numeric(boot_df['Winner Simple KM'],errors='ignore') -
pd.to_numeric(boot_df['Non-Winner Simple KM'], errors='ignore')
    nomwindiff = MSLT.confidence_interval(boot_df, 'Non-Winner Winner
Difference', 0.95)
    nomwinkm = MSLT.confidence_interval(boot_df, 'Non-Winner Winner
Difference KM', 0.95)
    MSMPointEstimates = [win_best, non_best, winnon_best]
    MSMConfIntervals = [win_msm, non_msm, nomwindiff]
    MSM_column = []
    for i in range(0, len(MSMPointEstimates)):
        MSM_column.append(str(MSMPointEstimates[i]) + " " +
str(MSMConfIntervals[i]))
    KMPointEstimates = [winKM_best, nonKM_best, winnonKM_best]
    KMConfIntervals = [win_km, non_km, nomwinkm]
    KM_column = []
    for i in range(0, len(KMPointEstimates)):
        KM_column.append(str(KMPointEstimates[i]) + " " +
str(KMConfIntervals[i]))
    index = ['Winner Life Expectancy', 'Non-Winner Life Expectancy',
'Winner Non-Winner Difference']
    sum_df = pd.DataFrame(list(zip(MSM_column, KM_column,
index)),columns=['Multi-State Modelling Method (Estimate [CI])', 'Kaplan
Meier Method (Estimate [CI])', 'Measure'])
    sum_df.set_index('Measure', inplace=True)
    sum_df.to_csv("C:/Users/NEVET/Desktop/Stars/python/" + outfile)
```

```
import MultiStateLifeTable as MSLT
import WinnerVsNonWinnerMSM as WIN
import pandas as pd
import numpy as np
import string
import matplotlib.pyplot as plt
import copy

# This file has codes to
# 1)  Do further data cleaning (same in every file starting with 2_)
# 2)  Produce life expectancies stratified by baseline characteristics
(Table2).
# Everyone is being treated as if they have only 4 possible statuses like
# controls, but you do not censor them at nomination or winning time.
# The following functions were taken from MultiStateLifeTable file and
modified:
#nomineewinnerlifeexpectancy
#bootstrap_life_ex
#confidence_interval
#summary_results


datafile = "OscarsDataMod.csv" # read in the data in expanded format ie.
including ages as well as dates from DataPrepMStat
df = pd.read_csv(datafile, names = ['Birth', 'First_Film',
'First_Ceremony', 'First_Nom', 'First_Win', 'Death', 'Final', 'Alive',
'Status', 'Male_1', \

'Born_White','Born_USA','Name_Change','Drama_Genre','Lead_1','TWins','TNom
s','MultiWinner','MultiNominee', \
                                    'final age', 'age of first film',
'first film status', 'age of celebration', 'celebration status', 'age at
death', \
                                    'death status', 'age of nomination',
'nomination status', 'age of victory', 'victory status'], skiprows=1)



df['victory status'][6085] = 0
df['age of victory'][6085] = float('NaN')
df['Status'][6085] = 'Nominee'
df['nomination status'][668] = 0
df['age of victory'][668] = float('NaN')
df['Status'][668] = 'Control'
df['nomination status'][139] = 0
df['age of victory'][139] = float('NaN')
df['Status'][139] = 'Control'
df['nomination status'][337] = 0
df['age of victory'][337] = float('NaN')
df['Status'][337] = 'Control'
df.index.name = 'Locator'
df['birth status'] = 1
df['age at birth'] = 0
```

```
df.loc[(df['Birth'] <= 1919), 'BirthEra'] = 0
df.loc[(df['Birth'] >= 1920), 'BirthEra'] = 1


transition_ages = {"person": "age at birth", "performer": "age of first
film", "celebrity": "age of celebration", "nominee": "age of nomination",
"winner": "age of victory", "dead": "age at death"}
transition_statuses = {"person": 'birth status', "performer": "first film
status", "celebrity": "celebration status", "nominee": "nomination
status", "winner": "victory status", "dead": "death status"}

states = transition_ages
statuses = transition_statuses



def covlifeexpectancy(data,transition_ages, transition_statuses, varx,
gp1, gp2):
    # INPUTS
    # "data": the data in expanded format with one performer per row -
this file is the output from datacleanup
    # "transition_ages": a dictionary matching each state to a column in
the data set showing what age the performer reaches the state at
    # (eg. {"person": "age at birth", "performer": "age of first film",
"nominee": "age of nomination", "dead": "age at death"})
    # "transition_statuses": a dictionary matching each state to a column
in the data set showing whether a performer ever reached a state
    # {"person": 'birth status', "performer": "first film status",
"nominee": "age of nomination", "dead": "death status"}

    statesCon = ['person', 'performer', 'celebrity', 'dead']
    allowable_transitionsCon = MSLT.allowed_transitions(statesCon)


    gp1 = data[data[varx] ==0]
    gp2 = data[data[varx] ==1]

    rowsTo_gp1 = MSLT.df_to_list(gp1)
    rowsTo_gp2 = MSLT.df_to_list(gp2)


    risktransTo_gp1 = MSLT.atrisk_and_transitions(transition_ages,
statesCon, transition_statuses, gp1, allowable_transitionsCon, rowsTo_gp1)
    risktransTo_gp2 = MSLT.atrisk_and_transitions(transition_ages,
statesCon, transition_statuses, gp2, allowable_transitionsCon, rowsTo_gp2)

    probsTo_gp1 = MSLT.transitionprobs_and_samplesizes(risktransTo_gp1[0],
risktransTo_gp1[1], allowable_transitionsCon, statesCon)
    probsTo_gp2 = MSLT.transitionprobs_and_samplesizes(risktransTo_gp2[0],
risktransTo_gp2[1], allowable_transitionsCon, statesCon)

    LifeEx_gp1 = MSLT.life_expectancy(probsTo_gp1[0], 'celebrity', 0,
statesCon)
```

```
    LifeEx_gp2 = MSLT.life_expectancy(probsTo_gp2[0], 'celebrity', 0,
statesCon)

    #print (LifeEx_gp1, LifeEx_gp2)
    return [LifeEx_gp1, LifeEx_gp2]

#covlifeexpectancy(df, transition_ages, transition_statuses, 'Male_1',
'Women', 'Men')
#covlifeexpectancy(df, transition_ages, transition_statuses, 'BirthEra')



def bootstrap_life_ex(data, transition_ages, transition_statuses, n,
outfile, varx, gp1, gp2):
    # INPUTS
    # "data": the data in expanded format with one performer per row -
this file is the output from datacleanup
    # "transition_ages": a dictionary matching each state to a column in
the data set showing what age the performer reaches the state at
    # (eg. {"person": "age at birth", "performer": "age of first film",
"nominee": "age of nomination", "dead": "age at death"})
    # "transition_statuses": a dictionary matching each state to a column
in the data set showing whether a performer ever reached a state
    # {"person": 'birth status', "performer": "first film status",
"nominee": "age of nomination", "dead": "death status"}
    # "n": the number of bootstraps to run
    # "outfile": the name of the file to output the bootstrap results to
(extension included)
    rows = []


    result = covlifeexpectancy (data, transition_ages,
transition_statuses, varx, gp1, gp2)
    rows.append(result)

    for i in range(0, len(rows[0])):
        rows[0][i] = round(rows[0][i], 1)
    print("BEST ESTIMATE")
    print(gp1+ " Life Expectancy: " + str(result[0]))
    print(gp2 + " Life Expectancy: " + str(result[1]))

    for i in range(0, n):
        boot_df = data.sample(frac = 1, replace = True)
        result = covlifeexpectancy(boot_df, transition_ages,
transition_statuses, varx, gp1, gp2)
        rows.append(result)
        print("BOOT RUN " + str(i+1))
        print(gp1 + " Life Expectancy: " + str(result[0]))
        print(gp2 + " Life Expectancy: " + str(result[1]))
    boot_results = pd.DataFrame(rows, columns=[gp1 + ' Life Expectancy',
gp2 + ' Life Expectancy'])
    boot_results.index.name = "Bootstrap Run"
    boot_results.rename(index={0: 'Best Estimate'}, inplace=True)
    boot_results.to_csv(outfile)
```

```
def confidence_interval(data, column_name, confidence_level):
    # INPUTS
    # "data": the bootstrap results imported as a dataframe (eg. using
pd.read_csv). precisely the output file from bootstrap_life_ex
    # "column_name": the statistic that you want the interval for,
specified by the name of the column containing it (eg. "Nominee Life
Expectancy")
    # "confidence_level": a real number between 0 and 1 that represents
the desired confidence level. eg. 0.95 for 95%
    results = data[column_name].tolist()
    results.sort()
    lower_bound = int((1 - confidence_level) / 2 * len(results)) - 1
    upper_bound = int((confidence_level + 1) / 2 * len(results)) - 1
    if lower_bound < 0:
        lower_bound = 0
    return [round(float(results[lower_bound]), 1),
round(float(results[upper_bound]), 1)]


def summary_results(bootstrap, outfile, gp1, gp2):
    # INPUTS
    # "bootstrap": the bootstrap results imported as a dataframe (eg.
using pd.read_csv). precisely the output file from bootstrap_life_ex
    # "outfile": the name of the file to output the summary of the
bootstrap results to (extension included)

    print (bootstrap)

    gp1_best = float(bootstrap[(gp1 + " Life Expectancy")]['Best
Estimate'])
    gp2_best = float(bootstrap[gp2 + " Life Expectancy"]['Best Estimate'])

    gp1_gp2_best = round(gp1_best - gp2_best, 1)
    boot_df = bootstrap.drop(['Best Estimate', 'Bootstrap Run'])
    boot_df.index.name = 'Bootstrap Run'
    gp1_msm = confidence_interval(boot_df, gp1 + ' Life Expectancy', 0.95)
    gp2_msm = confidence_interval(boot_df, gp2 + ' Life Expectancy', 0.95)

    boot_df[gp1 +gp2 + ' Difference'] = pd.to_numeric(boot_df[gp1 + ' Life
Expectancy'], errors='ignore') - pd.to_numeric(boot_df[gp2 + ' Life
Expectancy'], errors='ignore')
    gp1_gp2_diff = confidence_interval(boot_df, gp1 +gp2 + ' Difference',
0.95)

    MSMPointEstimates = [gp1_best, gp2_best, gp1_gp2_best]
    MSMConfIntervals = [gp1_msm, gp2_msm, gp1_gp2_diff]
    MSM_column = []
    for i in range(0, len(MSMPointEstimates)):
        MSM_column.append(str(MSMPointEstimates[i]) + " " +
str(MSMConfIntervals[i]))
```

```
    index = [gp1 + ' Life Expectancy', gp2 + ' Life Expectancy', gp1 + gp2
+ ' Difference']
    sum_df = pd.DataFrame(list(zip(MSM_column, index)),columns=['Multi-
State Modelling Method (Estimate [CI])', 'Measure'])
    sum_df.set_index('Measure', inplace=True)
    sum_df.to_csv(outfile)



bootstrap_life_ex(df, states, statuses, 1000,
"LifeExpectancyBootstrapTest_1.csv", 'BirthEra',  'Remote', 'Recent')
boot_file = pd.read_csv("LifeExpectancyBootstrapTest_1.csv", names =
['Remote Life Expectancy', 'Recent Life Expectancy'])
summary_results(boot_file, "SummaryResultsTest_1.csv", 'Remote', 'Recent')

bootstrap_life_ex(df, states, statuses, 1000,
"LifeExpectancyBootstrapTest_2.csv", 'Male_1',  'Women', 'Men')
boot_file = pd.read_csv("LifeExpectancyBootstrapTest_2.csv", names =
['Women Life Expectancy', 'Men Life Expectancy'])
summary_results(boot_file, "SummaryResultsTest_2.csv", 'Women', 'Men')


bootstrap_life_ex(df, states, statuses, 1000,
"LifeExpectancyBootstrapTest_3.csv", 'Born_White',  'Non-white', 'White')
boot_file = pd.read_csv("LifeExpectancyBootstrapTest_3.csv", names =
['Non-white Life Expectancy', 'White Life Expectancy'])
summary_results(boot_file, "SummaryResultsTest_3.csv", 'Non-white',
'White')

bootstrap_life_ex(df, states, statuses, 1000,
"LifeExpectancyBootstrapTest_4.csv", 'Born_USA',  'Non-USA', 'USA')
boot_file = pd.read_csv("LifeExpectancyBootstrapTest_4.csv", names =
['Non-USA Life Expectancy', 'USA Life Expectancy'])
summary_results(boot_file, "SummaryResultsTest_4.csv", 'Non-USA', 'USA')

bootstrap_life_ex(df, states, statuses, 1000,
"LifeExpectancyBootstrapTest_5.csv", 'Name_Change',  'Unchanged',
'Changed')
boot_file = pd.read_csv("LifeExpectancyBootstrapTest_5.csv", names =
['Unchanged Life Expectancy', 'Changed Life Expectancy'])
summary_results(boot_file, "SummaryResultsTest_5.csv", 'Unchanged',
'Changed')

bootstrap_life_ex(df, states, statuses, 1000,
"LifeExpectancyBootstrapTest_6.csv", 'Drama_Genre',  'Any other', 'Drama')
boot_file = pd.read_csv("LifeExpectancyBootstrapTest_6.csv", names = ['Any
other Life Expectancy', 'Drama Life Expectancy'])
summary_results(boot_file, "SummaryResultsTest_6.csv", 'Any other',
'Drama')
```

```
import MultiStateLifeTable as MSLT
import WinnerVsNonWinnerMSM as WIN
import pandas as pd
import numpy as np
import string
import matplotlib.pyplot as plt
import copy


# This file has codes to
   1) Do further data cleaning (same in every file starting with 2_)
   2) Create dataset to draw survival curves in Excel (Figure1)
# The function "nomineewinnerlifeexpectancy" is taken from
MultiStateLifeTable and modified slightly to output survival rates



datafile = "OscarsDataMod.csv" # read in the data in expanded format ie.
including ages as well as dates from DataPrepMStat
df = pd.read_csv(datafile, names = ['Birth', 'First_Film',
'First_Ceremony', 'First_Nom', 'First_Win', 'Death', 'Final', 'Alive',
'Status', 'Male_1', \

'Born_White','Born_USA','Name_Change','Drama_Genre','Lead_1','TWins','TNo
ms','MultiWinner','MultiNominee', \
                                    'final age', 'age of first film',
'first film status', 'age of celebration', 'celebration status', 'age at
death', \
                                    'death status', 'age of nomination',
'nomination status', 'age of victory', 'victory status'], skiprows=1)



df['victory status'][6085] = 0
df['age of victory'][6085] = float('NaN')
df['Status'][6085] = 'Nominee'
df['nomination status'][668] = 0
df['age of victory'][668] = float('NaN')
df['Status'][668] = 'Control'
df['nomination status'][139] = 0
df['age of victory'][139] = float('NaN')
df['Status'][139] = 'Control'
df['nomination status'][337] = 0
df['age of victory'][337] = float('NaN')
df['Status'][337] = 'Control'
df.index.name = 'Locator'
df['birth status'] = 1
df['age at birth'] = 0


transition_ages = {"person": "age at birth", "performer": "age of first
film", "celebrity": "age of celebration", "nominee": "age of nomination",
"winner": "age of victory", "dead": "age at death"}
```

```
transition_statuses = {"person": 'birth status', "performer": "first film status", "celebrity": "celebration status", "nominee": "nomination status", "winner": "victory status", "dead": "death status"}

states = transition_ages
statuses = transition_statuses




def nomineewinnerlifeexpectancy(data, transition_ages, transition_statuses):
    # INPUTS
    # "data": the data in expanded format with one performer per row - this file is the output from datacleanup
    # "transition_ages": a dictionary matching each state to a column in the data set showing what age the performer reaches the state at
    # (eg. {"person": "age at birth", "performer": "age of first film", "nominee": "age of nomination", "dead": "age at death"})
    # "transition_statuses": a dictionary matching each state to a column in the data set showing whether a performer ever reached a state
    # {"person": 'birth status', "performer": "first film status", "nominee": "age of nomination", "dead": "death status"}
    states = ['person', 'performer', 'celebrity', 'nominee', 'winner', 'dead']
    statesNom = ['person', 'performer', 'celebrity', 'nominee', 'dead']
    statesCon = ['person', 'performer', 'celebrity', 'dead']

    rowsFull = MSLT.df_to_list(data)
    rowsToNom = MSLT.censor_at_winner(data, rowsFull)
    rowsToCon = MSLT.censor_at_nominee(data, rowsFull)

    allowable_transitions = MSLT.allowed_transitions(states)
    allowable_transitionsNom = MSLT.allowed_transitions(statesNom)
    allowable_transitionsCon = MSLT.allowed_transitions(statesCon)

    risktransFull = MSLT.atrisk_and_transitions(transition_ages, states, transition_statuses, data, allowable_transitions, rowsFull)
    risktransToNom = MSLT.atrisk_and_transitions(transition_ages, statesNom, transition_statuses, data, allowable_transitionsNom, rowsToNom)
    risktransToCon = MSLT.atrisk_and_transitions(transition_ages, statesCon, transition_statuses, data, allowable_transitionsCon, rowsToCon)

    riskW = risktransFull[0]
    transW = risktransFull[1]

    riskN = risktransToNom[0]
    transN = risktransToNom[1]

    riskC  = risktransToCon[0]
    transC = risktransToCon[1]
```

```
    riskW.to_csv("C:/Users/NEVET/Desktop/Stars/python/riskW.csv")
    transW.to_csv("C:/Users/NEVET/Desktop/Stars/python/transW.csv")

    riskN.to_csv("C:/Users/NEVET/Desktop/Stars/python/riskN.csv")
    transN.to_csv("C:/Users/NEVET/Desktop/Stars/python/transN.csv")

    riskC.to_csv("C:/Users/NEVET/Desktop/Stars/python/riskC.csv")
    transC.to_csv("C:/Users/NEVET/Desktop/Stars/python/transC.csv")

    probsFull = MSLT.transitionprobs_and_samplesizes(risktransFull[0],
risktransFull[1], allowable_transitions, states)
    probsToNom = MSLT.transitionprobs_and_samplesizes(risktransToNom[0],
risktransToNom[1], allowable_transitionsNom, statesNom)
    probsToCon = MSLT.transitionprobs_and_samplesizes(risktransToCon[0],
risktransToCon[1], allowable_transitionsCon, statesCon)

    radixFull = np.array([[0],[0],[0],[0],[1],[0]], dtype=float)
    radixToNom = np.array([[0],[0],[0],[1],[0]], dtype=float)
    radixToCon = np.array([[0], [0], [1], [0]], dtype=float)

    survFull = MSLT.survivorship_vector(probsFull[0], radixFull, 0,
states)
    survToNom = MSLT.survivorship_vector(probsToNom[0], radixToNom, 0,
statesNom)
    survToCon = MSLT.survivorship_vector(probsToCon[0], radixToCon, 0,
statesCon)

    survFull.to_csv("C:/Users/NEVET/Desktop/Stars/python/SurvW.csv")
    survToNom.to_csv("C:/Users/NEVET/Desktop/Stars/python/SurvN.csv")
    survToCon.to_csv("C:/Users/NEVET/Desktop/Stars/python/SurvC.csv")


    nomLifeEx = MSLT.life_expectancy(probsToNom[0], 'nominee', 0,
statesNom)
    winLifeEx = MSLT.life_expectancy(probsFull[0], 'winner', 0, states)
    conLifeEx = MSLT.life_expectancy(probsToCon[0], 'celebrity', 0,
statesCon)

    print (nomLifeEx, winLifeEx, conLifeEx)
    return [nomLifeEx, winLifeEx, conLifeEx]


nomineewinnerlifeexpectancy(df, transition_ages, transition_statuses)
```

```
import MultiStateLifeTable as MSLT
import WinnerVsNonWinnerMSM as WIN
import pandas as pd
import numpy as np
import string
import matplotlib.pyplot as plt
import copy


# This file has codes to
# 1)  Do further data cleaning (same in every file with starting 2_)
# 2)  Produce life expectancies for controls, nominees and winners within
each subgroup (Table3).
# Controls include nominees and winners up to nomination year
# Winners include only winners
# Nominees include nominees and winners up to winning year

# The following functions were taken from MultiStateLifeTable file and
modified:
#nomineewinnerlifeexpectancy
#bootstrap_life_ex
#confidence_interval
#summary_results


datafile = "OscarsDataMod.csv" # read in the data in expanded format ie.
including ages as well as dates from DataPrepMStat
df = pd.read_csv(datafile, names = ['Birth', 'First_Film',
'First_Ceremony', 'First_Nom', 'First_Win', 'Death', 'Final', 'Alive',
'Status', 'Male_1', \

'Born_White','Born_USA','Name_Change','Drama_Genre','Lead_1','TWins','TNo
ms','MultiWinner','MultiNominee', \
                                    'final age', 'age of first film',
'first film status', 'age of celebration', 'celebration status', 'age at
death', \
                                    'death status', 'age of nomination',
'nomination status', 'age of victory', 'victory status'], skiprows=1)



df['victory status'][6085] = 0
df['age of victory'][6085] = float('NaN')
df['Status'][6085] = 'Nominee'
df['nomination status'][668] = 0
df['age of victory'][668] = float('NaN')
df['Status'][668] = 'Control'
df['nomination status'][139] = 0
df['age of victory'][139] = float('NaN')
df['Status'][139] = 'Control'
df['nomination status'][337] = 0
df['age of victory'][337] = float('NaN')
df['Status'][337] = 'Control'
```

```
df.index.name = 'Locator'
df['birth status'] = 1
df['age at birth'] = 0
```

```
df['IncludeAll'] = 1

# Remote birth era vs recent birth era
df.loc[(df['Birth'] <= 1919), 'BirthEra'] = 0
df.loc[(df['Birth'] >= 1920), 'BirthEra'] = 1

# Single win vs multiple wins
df.loc[(df['MultiWinner']==0) & (df['Status']=='Winner'), 'MultiWin'] = 0
df.loc[(df['MultiWinner']==1) & (df['Status']=='Winner'), 'MultiWin'] = 1


# Younger win vs older win
df.loc[(df['age of victory'] <40) & (df['Status']=='Winner'), 'OldWin'] =
0
df.loc[(df['age of victory'] >= 40) & (df['Status']=='Winner'), 'OldWin']
= 1

# Leading role vs supporting role
df.loc[(df['Lead_1']==0) & (df['Status']=='Winner'), 'LeadWin'] = 0
df.loc[(df['Lead_1']==1) & (df['Status']=='Winner'), 'LeadWin'] = 1


# Single nominiation vs multiple nominations
df.loc[(df['MultiNominee']==0) & (df['Status'] != 'Control'), 'MultiNom']
= 0
df.loc[(df['MultiNominee']==1) & (df['Status'] != 'Control'), 'MultiNom']
= 1

transition_ages = {"person": "age at birth", "performer": "age of first
film", "celebrity": "age of celebration", "nominee": "age of nomination",
"winner": "age of victory", "dead": "age at death"}
transition_statuses = {"person": 'birth status', "performer": "first film
status", "celebrity": "celebration status", "nominee": "nomination
status", "winner": "victory status", "dead": "death status"}

states = transition_ages
statuses = transition_statuses


countx = df.groupby(['BirthEra','Status']).size()
print (countx)

countx = df.groupby(['Male_1','Status']).size()
print (countx)
```

```
countx = df.groupby(['Born_White','Status']).size()
print (countx)


countxx = df['Status'].value_counts()
print (countxx)



def nomineewinnerlifeexpectancy(data, transition_ages,
transition_statuses):
    # INPUTS
    # "data": the data in expanded format with one performer per row -
this file is the output from datacleanup
    # "transition_ages": a dictionary matching each state to a column in
the data set showing what age the performer reaches the state at
    # (eg. {"person": "age at birth", "performer": "age of first film",
"nominee": "age of nomination", "dead": "age at death"})
    # "transition_statuses": a dictionary matching each state to a column
in the data set showing whether a performer ever reached a state
    # {"person": 'birth status', "performer": "first film status",
"nominee": "age of nomination", "dead": "death status"}
    states = ['person', 'performer', 'celebrity', 'nominee', 'winner',
'dead']
    statesNom = ['person', 'performer', 'celebrity', 'nominee', 'dead']
    statesCon = ['person', 'performer', 'celebrity', 'dead']

    rowsFull  = MSLT.df_to_list(data)
    rowsToNom = MSLT.censor_at_winner(data, rowsFull)
    rowsToCon = MSLT.censor_at_nominee(data, rowsFull)

    allowable_transitions = MSLT.allowed_transitions(states)
    allowable_transitionsNom = MSLT.allowed_transitions(statesNom)
    allowable_transitionsCon = MSLT.allowed_transitions(statesCon)

    risktransFull = MSLT.atrisk_and_transitions(transition_ages, states,
transition_statuses, data, allowable_transitions, rowsFull)
    risktransToNom = MSLT.atrisk_and_transitions(transition_ages,
statesNom, transition_statuses, data, allowable_transitionsNom,
rowsToNom)
    risktransToCon = MSLT.atrisk_and_transitions(transition_ages,
statesCon, transition_statuses, data, allowable_transitionsCon,
rowsToCon)

    probsFull = MSLT.transitionprobs_and_samplesizes(risktransFull[0],
risktransFull[1], allowable_transitions, states)
    probsToNom = MSLT.transitionprobs_and_samplesizes(risktransToNom[0],
risktransToNom[1], allowable_transitionsNom, statesNom)
    probsToCon = MSLT.transitionprobs_and_samplesizes(risktransToCon[0],
risktransToCon[1], allowable_transitionsCon, statesCon)

    nomLifeEx = MSLT.life_expectancy(probsToNom[0], 'nominee', 0,
statesNom)
    winLifeEx = MSLT.life_expectancy(probsFull[0], 'winner', 0, states)
```

```
    conLifeEx = MSLT.life_expectancy(probsToCon[0], 'celebrity', 0,
statesCon)
    return [nomLifeEx, winLifeEx, conLifeEx]


def bootstrap_life_ex(data, transition_ages, transition_statuses, n,
outfile, varx, val, gp):
    # INPUTS
    # "data": the data in expanded format with one performer per row -
this file is the output from datacleanup
    # "transition_ages": a dictionary matching each state to a column in
the data set showing what age the performer reaches the state at
    # (eg. {"person": "age at birth", "performer": "age of first film",
"nominee": "age of nomination", "dead": "age at death"})
    # "transition_statuses": a dictionary matching each state to a column
in the data set showing whether a performer ever reached a state
    # {"person": 'birth status', "performer": "first film status",
"nominee": "age of nomination", "dead": "death status"}
    # "n": the number of bootstraps to run
    # "outfile": the name of the file to output the bootstrap results to
(extension included)
    rows = []

    result = nomineewinnerlifeexpectancy(data, transition_ages,
transition_statuses)
    rows.append(result)

    print ('Number of stars in ',gp, varx)
    print (data.shape[0])
    count = data['Status'].value_counts()
    print (count)

    for i in range(0, len(rows[0])):
        rows[0][i] = round(rows[0][i], 1)
    #print("BEST ESTIMATE")
    #print("Nominee Life Expectancy: " + str(result[0]))
    #print("Winner Life Expectancy: " + str(result[1]))
    #print("Control Life Expectancy: "+ str(result[2]))

    for i in range(0, n):
        boot_df = data.sample(frac = 1, replace = True)

        #print ('Number of stars in bootstrap sample',gp, varx)
        #print (boot_df.shape[0])
        #countx = boot_df['Status'].value_counts()
        #print (countx)

        result = nomineewinnerlifeexpectancy(boot_df, transition_ages,
transition_statuses)
        rows.append(result)

        print("BOOT RUN " + str(i+1))
        #print("Nominee Life Expectancy: " + str(result[0]))
        #print("Winner Life Expectancy: " + str(result[1]))
```

```
        #print("Control Life Expectancy: " + str(result[2]))

    boot_results = pd.DataFrame(rows, columns=[varx + ": " + gp + 
'Nominee', varx + ": " + gp + 'Winner', varx + ": " + gp + 'Control'])
    boot_results.index.name = "Bootstrap Run"
    boot_results.rename(index={0: 'Best Estimate'}, inplace=True)
    boot_results.to_csv(outfile)
    return [boot_results]

def confidence_interval(data, column_name, confidence_level):
    # INPUTS
    # "data": the bootstrap results imported as a dataframe (eg. using 
pd.read_csv). precisely the output file from bootstrap_life_ex
    # "column_name": the statistic that you want the interval for, 
specified by the name of the column containing it (eg. "Nominee Life 
Expectancy")
    # "confidence_level": a real number between 0 and 1 that represents 
the desired confidence level. eg. 0.95 for 95%
    results = data[column_name].tolist()
    results.sort()
    lower_bound = int((1 - confidence_level) / 2 * len(results)) - 1
    upper_bound = int((confidence_level + 1) / 2 * len(results)) - 1
    if lower_bound < 0:
        lower_bound = 0
    return [round(float(results[lower_bound]), 1), 
round(float(results[upper_bound]), 1)]


def summary_results(bootstrap, outfile, varx, val, gp):
    # INPUTS
    # "bootstrap": the bootstrap results imported as a dataframe (eg. 
using pd.read_csv). precisely the output file from bootstrap_life_ex
    # "outfile": the name of the file to output the summary of the 
bootstrap results to (extension included)
    nom_best = float(bootstrap[varx + ": " + gp + 'Nominee']['Best 
Estimate'])
    win_best = float(bootstrap[varx + ": " + gp + 'Winner']['Best 
Estimate'])
    con_best = float(bootstrap[varx + ": " + gp + 'Control']['Best 
Estimate'])

    nomwin_best = round(win_best - nom_best, 1)
    wincon_best = round(win_best - con_best, 1)
    nomcon_best = round(nom_best - con_best, 1)

    boot_df = bootstrap.drop(['Best Estimate', 'Bootstrap Run'])
    boot_df.index.name = 'Bootstrap Run'

    nom_msm = confidence_interval(boot_df, varx + ": " + gp + 'Nominee', 
0.95)
    win_msm = confidence_interval(boot_df, varx + ": " + gp + 'Winner', 
0.95)
    con_msm = confidence_interval(boot_df, varx + ": " + gp + 'Control', 
0.95)
```

```
    boot_df['Nominee Winner Difference'] = pd.to_numeric(boot_df[varx +
": " + gp + 'Winner'], errors='ignore') - pd.to_numeric(boot_df[varx + ":
" + gp + 'Nominee'], errors='ignore')
    boot_df['Control Winner Difference'] = pd.to_numeric(boot_df[varx +
": " + gp + 'Winner'], errors='ignore') - pd.to_numeric(boot_df[varx + ":
" + gp + 'Control'], errors='ignore')
    boot_df['Nominee Control Difference'] = pd.to_numeric(boot_df[varx +
": " + gp + 'Nominee'], errors='ignore') - pd.to_numeric(boot_df[varx +
": " + gp + 'Control'], errors='ignore')

    nomwindiff = confidence_interval(boot_df, 'Nominee Winner
Difference', 0.95)
    conwindiff = confidence_interval(boot_df, 'Control Winner
Difference', 0.95)
    nomcondiff = confidence_interval(boot_df, 'Nominee Control
Difference', 0.95)

    MSMPointEstimates = [win_best, nom_best, con_best, nomwin_best,
wincon_best, nomcon_best]
    MSMConfIntervals = [win_msm, nom_msm, con_msm, nomwindiff,
conwindiff, nomcondiff]

    MSM_column = []
    for i in range(0, len(MSMPointEstimates)):
        MSM_column.append(str(MSMPointEstimates[i]) + " " +
str(MSMConfIntervals[i]))

    index = ['Winner', 'Nominee', 'Control','Winner-Nominee Difference',
'Winner-Control Difference', 'Nominee-Control Difference']
    sum_df = pd.DataFrame(list(zip(MSM_column, index)),columns=[(varx +
": " + gp), 'Measure'])
    sum_df.set_index('Measure', inplace=True)
    sum_df.to_csv(outfile)
    return [sum_df]



def repeat(all_data, varx, val, gp):
    sub_df = all_data[all_data[varx] == val]
    bootstrap_life_ex(sub_df, states, statuses, 1000,
varx+str(val)+"_"+gp+"_"+"LE.csv",varx, val, gp)
    boot_file = pd.read_csv(varx+str(val)+"_"+gp+"_"+"LE.csv", names =
[varx + ": " + gp + 'Nominee', varx + ": " + gp + 'Winner', varx + ": " +
gp + 'Control'])
    #df[varx + ": " + gp + 'Nominee', varx + ": " + gp + 'Winner', varx +
": " + gp + 'Control'] = boot_file
    summary_results(boot_file, varx+str(val)+"_"+gp+"_"+"Summary.csv",
varx, val, gp)
    #boot_file.columns=['a','b','c']
    #index = ['Winner Life Expectancy', 'Nominee Life Expectancy',
'Control Life Expectancy','Winner-Nominee Difference', 'Winner-Control
Difference', 'Nominee-Control Difference']
```

```
    #Summary = pd.DataFrame(list(zip(sumx, index)),columns=[(varx+ ": " +
gp), 'Measure'])
    #LE = pd.DataFrame(, columns=['Nominee Life Expectancy', 'Winner Life
Expectancy', 'Control Life Expectancy'])
    #LE.append(boot_file)



# Table 3A;
repeat(df, 'IncludeAll', 1, 'All')

#repeat(df, 'BirthEra', 0, 'Remote')
#repeat(df, 'BirthEra', 1, 'Recent')

#repeat(df, 'Male_1', 0, 'Female')
#repeat(df, 'Male_1', 1, 'Male')

#repeat(df, 'Born_White', 0, 'Non_white')
#repeat(df, 'Born_White', 1, 'White')


# Table 3B;
repeat(df, 'MultiWin', 0, 'SingleWin')
repeat(df, 'MultiWin', 1, 'MultiWin')

repeat(df, 'OldWin', 0, 'YoungWin')
repeat(df, 'OldWin', 1, 'OldWin')

repeat(df, 'LeadWin', 0, 'SupportingRole')
repeat(df, 'LeadWin', 1, 'LeadRole')


# Table 3C;
repeat(df, 'MultiNom', 0, 'SingleNom1')
repeat(df, 'MultiNom', 1, 'MultiNom1')
```

```
import MultiStateLifeTable as MSLT
import WinnerVsNonWinnerMSM as WIN
import pandas as pd
import numpy as np
import string
import matplotlib.pyplot as plt
import copy


# This file has codes to
# 1)  Do further data cleaning (same in every file with starting 2_)
# 2)  Produce life expectancies for controls, nominees and winners within
each subgroup (Table3prime).
# Controls include nominees and winners up to nomination year
# Winners include only winners
# Nominees include only nominees (In Table 3, nominees also include
winners up to winning year)

# The following functions were taken from MultiStateLifeTable file and
modified:
#nomineewinnerlifeexpectancy
#bootstrap_life_ex
#confidence_interval
#summary_results


datafile = "OscarsDataMod.csv" # read in the data in expanded format ie.
including ages as well as dates from DataPrepMStat
df = pd.read_csv(datafile, names = ['Birth', 'First_Film',
'First_Ceremony', 'First_Nom', 'First_Win', 'Death', 'Final', 'Alive',
'Status', 'Male_1', \

'Born_White','Born_USA','Name_Change','Drama_Genre','Lead_1','TWins','TNo
ms','MultiWinner','MultiNominee', \
                                    'final age', 'age of first film',
'first film status', 'age of celebration', 'celebration status', 'age at
death', \
                                    'death status', 'age of nomination',
'nomination status', 'age of victory', 'victory status'], skiprows=1)



df['victory status'][6085] = 0
df['age of victory'][6085] = float('NaN')
df['Status'][6085] = 'Nominee'
df['nomination status'][668] = 0
df['age of victory'][668] = float('NaN')
df['Status'][668] = 'Control'
df['nomination status'][139] = 0
df['age of victory'][139] = float('NaN')
df['Status'][139] = 'Control'
df['nomination status'][337] = 0
df['age of victory'][337] = float('NaN')
```

```
df['Status'][337] = 'Control'
df.index.name = 'Locator'
df['birth status'] = 1
df['age at birth'] = 0
```

```
# Single nominiation vs multiple nominations - nominee groups include
only nominees!
df.loc[(df['MultiNominee']==0) & (df['Status'] == 'Nominee'), 'MultiNom']
= 0
df.loc[(df['MultiNominee']==1) & (df['Status'] == 'Nominee'), 'MultiNom']
= 1
df.loc[(df['Status'] == 'Nominee'), 'IncludeAll'] = 1
```


```
# Single nominiation vs multiple nominations - nominee groups include
only nominees!
df.loc[(df['MultiNominee']==0) & (df['Status'] == 'Nominee'), 'MultiNom']
= 0
df.loc[(df['MultiNominee']==1) & (df['Status'] == 'Nominee'), 'MultiNom']
= 1
df.loc[(df['Status'] == 'Nominee'), 'IncludeAll'] = 1
```


```
# Younger nominee vs older nominee - only nominees
df.loc[(df['age of nomination'] <40) & (df['Status']=='Nominee'),
'OldNom'] = 0
df.loc[(df['age of nomination'] >= 40) & (df['Status']=='Nominee'),
'OldNom'] = 1

# Leading role vs supporting role - only nominees
df.loc[(df['Lead_1']==0) & (df['Status']=='Nominee'), 'LeadNom'] = 0
df.loc[(df['Lead_1']==1) & (df['Status']=='Nominee'), 'LeadNom'] = 1


# Remote birth era vs recent birth era - only nominees
df.loc[(df['Birth'] <= 1919) & (df['Status']=='Nominee'), 'RecentNom'] =
0
df.loc[(df['Birth'] >= 1920) & (df['Status']=='Nominee'), 'RecentNom'] =
1


# Female nominees vs male nominees
df.loc[(df['Male_1']==0) & (df['Status']=='Nominee'), 'MaleNom'] = 0
df.loc[(df['Male_1']==1) & (df['Status']=='Nominee'), 'MaleNom'] = 1


# White nominees vs non-white nominees
df.loc[(df['Born_White']==0) & (df['Status']=='Nominee'), 'WhiteNom'] = 0
```

```
df.loc[(df['Born_White']==1) & (df['Status']=='Nominee'), 'WhiteNom'] = 1



countx = df.groupby(['MultiNom','Status']).size()
print (countx)

countxx = df['Status'].value_counts()
print (countxx)

transition_ages = {"person": "age at birth", "performer": "age of first
film", "celebrity": "age of celebration", "nominee": "age of nomination",
"winner": "age of victory", "dead": "age at death"}
transition_statuses = {"person": 'birth status', "performer": "first film
status", "celebrity": "celebration status", "nominee": "nomination
status", "winner": "victory status", "dead": "death status"}

states = transition_ages
statuses = transition_statuses

def nomineewinnerlifeexpectancy(data, transition_ages,
transition_statuses, varx, val, gp):
    # INPUTS
    # "data": the data in expanded format with one performer per row -
this file is the output from datacleanup
    # "transition_ages": a dictionary matching each state to a column in
the data set showing what age the performer reaches the state at
    # (eg. {"person": "age at birth", "performer": "age of first film",
"nominee": "age of nomination", "dead": "age at death"})
    # "transition_statuses": a dictionary matching each state to a column
in the data set showing whether a performer ever reached a state
    # {"person": 'birth status', "performer": "first film status",
"nominee": "age of nomination", "dead": "death status"}
    states = ['person', 'performer', 'celebrity', 'nominee', 'winner',
'dead']
    statesNom = ['person', 'performer', 'celebrity', 'nominee', 'dead']
    statesCon = ['person', 'performer', 'celebrity', 'dead']

    sub_data = data[data[varx] == val]

    rowsFull  = MSLT.df_to_list(data)

    rowsToNomList  = MSLT.df_to_list(sub_data)
    rowsToNom = MSLT.censor_at_winner(sub_data, rowsToNomList)

    rowsToCon = MSLT.censor_at_nominee(data, rowsFull)

    allowable_transitions = MSLT.allowed_transitions(states)
    allowable_transitionsNom = MSLT.allowed_transitions(statesNom)
    allowable_transitionsCon = MSLT.allowed_transitions(statesCon)

    risktransToWin = MSLT.atrisk_and_transitions(transition_ages, states,
transition_statuses, data, allowable_transitions, rowsFull)
```

```
    risktransToNom = MSLT.atrisk_and_transitions(transition_ages,
statesNom, transition_statuses, sub_data, allowable_transitionsNom,
rowsToNom)
    risktransToCon = MSLT.atrisk_and_transitions(transition_ages,
statesCon, transition_statuses, data, allowable_transitionsCon,
rowsToCon)

    probsFull = MSLT.transitionprobs_and_samplesizes(risktransToWin[0],
risktransToWin[1], allowable_transitions, states)
    probsToNom = MSLT.transitionprobs_and_samplesizes(risktransToNom[0],
risktransToNom[1], allowable_transitionsNom, statesNom)
    probsToCon = MSLT.transitionprobs_and_samplesizes(risktransToCon[0],
risktransToCon[1], allowable_transitionsCon, statesCon)

    nomLifeEx = MSLT.life_expectancy(probsToNom[0], 'nominee', 0,
statesNom)
    winLifeEx = MSLT.life_expectancy(probsFull[0], 'winner', 0, states)
    conLifeEx = MSLT.life_expectancy(probsToCon[0], 'celebrity', 0,
statesCon)
    return [nomLifeEx, winLifeEx, conLifeEx]


def bootstrap_life_ex(data, transition_ages, transition_statuses, n,
outfile, varx, val, gp):
    # INPUTS
    # "data": the data in expanded format with one performer per row -
this file is the output from datacleanup
    # "transition_ages": a dictionary matching each state to a column in
the data set showing what age the performer reaches the state at
    # (eg. {"person": "age at birth", "performer": "age of first film",
"nominee": "age of nomination", "dead": "age at death"})
    # "transition_statuses": a dictionary matching each state to a column
in the data set showing whether a performer ever reached a state
    # {"person": 'birth status', "performer": "first film status",
"nominee": "age of nomination", "dead": "death status"}
    # "n": the number of bootstraps to run
    # "outfile": the name of the file to output the bootstrap results to
(extension included)
    rows = []

    result = nomineewinnerlifeexpectancy(data, transition_ages,
transition_statuses, varx, val, gp)
    rows.append(result)

    subx = data[data[varx] == val]
    print (subx.shape[0])
    countx = subx['Status'].value_counts()
    print (countx)


    for i in range(0, len(rows[0])):
        rows[0][i] = round(rows[0][i], 1)
    #print("BEST ESTIMATE")
    #print("Nominee Life Expectancy: " + str(result[0]))
```

```
    #print("Winner Life Expectancy: " + str(result[1]))
    #print("Control Life Expectancy: "+ str(result[2]))

    for i in range(0, n):
        boot_df = data.sample(frac = 1, replace = True)
        result = nomineewinnerlifeexpectancy(boot_df, transition_ages,
transition_statuses, varx, val, gp)
        rows.append(result)
        print("BOOT RUN " + str(i+1))
        #print("Nominee Life Expectancy: " + str(result[0]))
        #print("Winner Life Expectancy: " + str(result[1]))
        #print("Control Life Expectancy: " + str(result[2]))

    boot_results = pd.DataFrame(rows, columns=[varx + ": " + gp +
'Nominee', varx + ": " + gp + 'Winner', varx + ": " + gp + 'Control'])
    boot_results.index.name = "Bootstrap Run"
    boot_results.rename(index={0: 'Best Estimate'}, inplace=True)
    boot_results.to_csv(outfile)
    return [boot_results]

def confidence_interval(data, column_name, confidence_level):
    # INPUTS
    # "data": the bootstrap results imported as a dataframe (eg. using
pd.read_csv). precisely the output file from bootstrap_life_ex
    # "column_name": the statistic that you want the interval for,
specified by the name of the column containing it (eg. "Nominee Life
Expectancy")
    # "confidence_level": a real number between 0 and 1 that represents
the desired confidence level. eg. 0.95 for 95%
    results = data[column_name].tolist()
    results.sort()
    lower_bound = int((1 - confidence_level) / 2 * len(results)) - 1
    upper_bound = int((confidence_level + 1) / 2 * len(results)) - 1
    if lower_bound < 0:
        lower_bound = 0
    return [round(float(results[lower_bound]), 1),
round(float(results[upper_bound]), 1)]


def summary_results(bootstrap, outfile, varx, val, gp):
    # INPUTS
    # "bootstrap": the bootstrap results imported as a dataframe (eg.
using pd.read_csv). precisely the output file from bootstrap_life_ex
    # "outfile": the name of the file to output the summary of the
bootstrap results to (extension included)
    nom_best = float(bootstrap[varx + ": " + gp + 'Nominee']['Best
Estimate'])
    win_best = float(bootstrap[varx + ": " + gp + 'Winner']['Best
Estimate'])
    con_best = float(bootstrap[varx + ": " + gp + 'Control']['Best
Estimate'])

    nomwin_best = round(win_best - nom_best, 1)
    wincon_best = round(win_best - con_best, 1)
```

```
    nomcon_best = round(nom_best - con_best, 1)

    boot_df = bootstrap.drop(['Best Estimate', 'Bootstrap Run'])
    boot_df.index.name = 'Bootstrap Run'

    nom_msm = confidence_interval(boot_df, varx + ": " + gp + 'Nominee',
0.95)
    win_msm = confidence_interval(boot_df, varx + ": " + gp + 'Winner',
0.95)
    con_msm = confidence_interval(boot_df, varx + ": " + gp + 'Control',
0.95)

    boot_df['Nominee Winner Difference'] = pd.to_numeric(boot_df[varx +
": " + gp + 'Winner'], errors='ignore') - pd.to_numeric(boot_df[varx + ":
" + gp + 'Nominee'], errors='ignore')
    boot_df['Control Winner Difference'] = pd.to_numeric(boot_df[varx +
": " + gp + 'Winner'], errors='ignore') - pd.to_numeric(boot_df[varx + ":
" + gp + 'Control'], errors='ignore')
    boot_df['Nominee Control Difference'] = pd.to_numeric(boot_df[varx +
": " + gp + 'Nominee'], errors='ignore') - pd.to_numeric(boot_df[varx +
": " + gp + 'Control'], errors='ignore')

    nomwindiff = confidence_interval(boot_df, 'Nominee Winner
Difference', 0.95)
    conwindiff = confidence_interval(boot_df, 'Control Winner
Difference', 0.95)
    nomcondiff = confidence_interval(boot_df, 'Nominee Control
Difference', 0.95)

    MSMPointEstimates = [win_best, nom_best, con_best, nomwin_best,
wincon_best, nomcon_best]
    MSMConfIntervals = [win_msm, nom_msm, con_msm, nomwindiff,
conwindiff, nomcondiff]

    MSM_column = []
    for i in range(0, len(MSMPointEstimates)):
        MSM_column.append(str(MSMPointEstimates[i]) + " " +
str(MSMConfIntervals[i]))

    index = ['Winner', 'Nominee', 'Control','Winner-Nominee Difference',
'Winner-Control Difference', 'Nominee-Control Difference']
    sum_df = pd.DataFrame(list(zip(MSM_column, index)),columns=[(varx +
": " + gp), 'Measure'])
    sum_df.set_index('Measure', inplace=True)
    sum_df.to_csv(outfile)
    return [sum_df]


def repeat(data_all, varx, val, gp):
    bootstrap_life_ex(data_all, states, statuses, 1000,
varx+str(val)+"_"+gp+"_"+"LE.csv",varx, val, gp)
    boot_file = pd.read_csv(varx+str(val)+"_"+gp+"_"+"LE.csv", names =
[varx + ": " + gp + 'Nominee', varx + ": " + gp + 'Winner', varx + ": " +
gp + 'Control'])
```

```
    summary_results(boot_file, varx+str(val)+"_"+gp+"_"+"Summary.csv",
varx, val, gp)


repeat(df, 'IncludeAll', 1, 'AllNominees2')

repeat(df, 'MultiNom', 0, 'SingleNom2')
repeat(df, 'MultiNom', 1, 'MultiNom2')

#repeat(df, 'OldNom', 0, 'YoungNom')
#repeat(df, 'OldNom', 1, 'OldNom')

#repeat(df, 'LeadNom', 0, 'SupportNom')
repeat(df, 'LeadNom', 1, 'LeadNom')

#repeat(df, 'RecentNom', 0, 'RemoteNom')
#repeat(df, 'RecentNom', 1, 'RecentNom')

#repeat(df, 'MaleNom', 0, 'FemaleNom')
#repeat(df, 'MaleNom', 1, 'MaleNom')

#repeat(df, 'WhiteNom', 0, 'NonWhiteNom')
#repeat(df, 'WhiteNom', 1, 'WhiteNom')
```

```
import MultiStateLifeTable as MSLT
import WinnerVsNonWinnerMSM as WIN
import pandas as pd
import numpy as np
import string
import matplotlib.pyplot as plt
import copy

# This file has codes to
1)    Do further data cleaning (same in every file starting 2_)
2)    Produce life expectancies for super subgroups (Table 4)
# The following functions were taken from MultiStateLifeTable file and
modified:
#nomineewinnerlifeexpectancy
#bootstrap_life_ex
#confidence_interval
#summary_results




datafile = "OscarsDataMod.csv" # read in the data in expanded format ie.
including ages as well as dates from DataPrepMStat
df = pd.read_csv(datafile, names = ['Birth', 'First_Film',
'First_Ceremony', 'First_Nom', 'First_Win', 'Death', 'Final', 'Alive',
'Status', 'Male_1', \

'Born_White','Born_USA','Name_Change','Drama_Genre','Lead_1','TWins','TNo
ms','MultiWinner','MultiNominee', \
                                    'final age', 'age of first film',
'first film status', 'age of celebration', 'celebration status', 'age at
death', \
                                    'death status', 'age of nomination',
'nomination status', 'age of victory', 'victory status'], skiprows=1)



df['victory status'][6085] = 0
df['age of victory'][6085] = float('NaN')
df['Status'][6085] = 'Nominee'
df['nomination status'][668] = 0
df['age of victory'][668] = float('NaN')
df['Status'][668] = 'Control'
df['nomination status'][139] = 0
df['age of victory'][139] = float('NaN')
df['Status'][139] = 'Control'
df['nomination status'][337] = 0
df['age of victory'][337] = float('NaN')
df['Status'][337] = 'Control'
df.index.name = 'Locator'
df['birth status'] = 1
df['age at birth'] = 0
```

```
# Remote birth era vs recent birth era
df.loc[(df['Birth'] <= 1919), 'BirthEra'] = 0
df.loc[(df['Birth'] >= 1920), 'BirthEra'] = 1

# Younger win vs older win
df.loc[(df['age of victory'] <40) & (df['Status']=='Winner'), 'OldWin'] =
0
df.loc[(df['age of victory'] >= 40) & (df['Status']=='Winner'), 'OldWin']
= 1

df['IncludeAll'] = 1


# super subgroups
df.loc[(df['BirthEra'] ==0) & (df['Male_1']==0) & (df['Born_White']==0),
'group'] = 1
df.loc[(df['BirthEra'] ==0) & (df['Male_1']==0) & (df['Born_White']==1),
'group'] = 2
df.loc[(df['BirthEra'] ==0) & (df['Male_1']==1) & (df['Born_White']==0),
'group'] = 3
df.loc[(df['BirthEra'] ==0) & (df['Male_1']==1) & (df['Born_White']==1),
'group'] = 4

df.loc[(df['BirthEra'] ==1) & (df['Male_1']==0) & (df['Born_White']==0),
'group'] = 5
df.loc[(df['BirthEra'] ==1) & (df['Male_1']==0) & (df['Born_White']==1),
'group'] = 6
df.loc[(df['BirthEra'] ==1) & (df['Male_1']==1) & (df['Born_White']==0),
'group'] = 7
df.loc[(df['BirthEra'] ==1) & (df['Male_1']==1) & (df['Born_White']==1),
'group'] = 8



transition_ages = {"person": "age at birth", "performer": "age of first
film", "celebrity": "age of celebration", "nominee": "age of nomination",
"winner": "age of victory", "dead": "age at death"}
transition_statuses = {"person": 'birth status', "performer": "first film
status", "celebrity": "celebration status", "nominee": "nomination
status", "winner": "victory status", "dead": "death status"}

states = transition_ages
statuses = transition_statuses


countx = df.groupby(['group','Status']).size()
print (countx)



def nomineewinnerlifeexpectancy(data, transition_ages,
transition_statuses):
    # INPUTS
```

```
    # "data": the data in expanded format with one performer per row - 
this file is the output from datacleanup
    # "transition_ages": a dictionary matching each state to a column in 
the data set showing what age the performer reaches the state at
    # (eg. {"person": "age at birth", "performer": "age of first film", 
"nominee": "age of nomination", "dead": "age at death"})
    # "transition_statuses": a dictionary matching each state to a column 
in the data set showing whether a performer ever reached a state
    # {"person": 'birth status', "performer": "first film status", 
"nominee": "age of nomination", "dead": "death status"}
    states = ['person', 'performer', 'celebrity', 'nominee', 'winner', 
'dead']
    statesNom = ['person', 'performer', 'celebrity', 'nominee', 'dead']
    statesCon = ['person', 'performer', 'celebrity', 'dead']

    rowsFull  = MSLT.df_to_list(data)
    rowsToNom = MSLT.censor_at_winner(data, rowsFull)
    rowsToCon = MSLT.censor_at_nominee(data, rowsFull)

    allowable_transitions = MSLT.allowed_transitions(states)
    allowable_transitionsNom = MSLT.allowed_transitions(statesNom)
    allowable_transitionsCon = MSLT.allowed_transitions(statesCon)

    risktransFull = MSLT.atrisk_and_transitions(transition_ages, states, 
transition_statuses, data, allowable_transitions, rowsFull)
    risktransToNom = MSLT.atrisk_and_transitions(transition_ages, 
statesNom, transition_statuses, data, allowable_transitionsNom, 
rowsToNom)
    risktransToCon = MSLT.atrisk_and_transitions(transition_ages, 
statesCon, transition_statuses, data, allowable_transitionsCon, 
rowsToCon)

    probsFull = MSLT.transitionprobs_and_samplesizes(risktransFull[0], 
risktransFull[1], allowable_transitions, states)
    probsToNom = MSLT.transitionprobs_and_samplesizes(risktransToNom[0], 
risktransToNom[1], allowable_transitionsNom, statesNom)
    probsToCon = MSLT.transitionprobs_and_samplesizes(risktransToCon[0], 
risktransToCon[1], allowable_transitionsCon, statesCon)

    nomLifeEx = MSLT.life_expectancy(probsToNom[0], 'nominee', 0, 
statesNom)
    winLifeEx = MSLT.life_expectancy(probsFull[0], 'winner', 0, states)
    conLifeEx = MSLT.life_expectancy(probsToCon[0], 'celebrity', 0, 
statesCon)

    print (risktransFull[1])
    print (winLifeEx)
    return [nomLifeEx, winLifeEx, conLifeEx]


def bootstrap_life_ex(data, transition_ages, transition_statuses, n, 
outfile, varx, val, gp):
    # INPUTS
```

```
    # "data": the data in expanded format with one performer per row - 
this file is the output from datacleanup
    # "transition_ages": a dictionary matching each state to a column in 
the data set showing what age the performer reaches the state at
    # (eg. {"person": "age at birth", "performer": "age of first film", 
"nominee": "age of nomination", "dead": "age at death"})
    # "transition_statuses": a dictionary matching each state to a column 
in the data set showing whether a performer ever reached a state
    # {"person": 'birth status', "performer": "first film status", 
"nominee": "age of nomination", "dead": "death status"}
    # "n": the number of bootstraps to run
    # "outfile": the name of the file to output the bootstrap results to 
(extension included)
    rows = []

    result = nomineewinnerlifeexpectancy(data, transition_ages, 
transition_statuses)
    rows.append(result)

    print ('Number of stars in ',gp, varx)
    print (data.shape[0])
    count = data['Status'].value_counts()
    print (count)

    for i in range(0, len(rows[0])):
        rows[0][i] = round(rows[0][i], 1)
    print("BEST ESTIMATE")
    print("Nominee Life Expectancy: " + str(result[0]))
    print("Winner Life Expectancy: " + str(result[1]))
    print("Control Life Expectancy: "+ str(result[2]))

    for i in range(0, n):
        boot_df = data.sample(frac = 1, replace = True)

        #print ('Number of stars in bootstrap sample',gp, varx)
        #print (boot_df.shape[0])
        #countx = boot_df['Status'].value_counts()
        #print (countx)

        result = nomineewinnerlifeexpectancy(boot_df, transition_ages, 
transition_statuses)
        rows.append(result)

        print("BOOT RUN " + str(i+1))
        #print("Nominee Life Expectancy: " + str(result[0]))
        #print("Winner Life Expectancy: " + str(result[1]))
        #print("Control Life Expectancy: " + str(result[2]))

    boot_results = pd.DataFrame(rows, columns=[varx + ": " + gp + 
'Nominee', varx + ": " + gp + 'Winner', varx + ": " + gp + 'Control'])
    boot_results.index.name = "Bootstrap Run"
    boot_results.rename(index={0: 'Best Estimate'}, inplace=True)
    boot_results.to_csv(outfile)
    return [boot_results]
```

```
def confidence_interval(data, column_name, confidence_level):
    # INPUTS
    # "data": the bootstrap results imported as a dataframe (eg. using
pd.read_csv). precisely the output file from bootstrap_life_ex
    # "column_name": the statistic that you want the interval for,
specified by the name of the column containing it (eg. "Nominee Life
Expectancy")
    # "confidence_level": a real number between 0 and 1 that represents
the desired confidence level. eg. 0.95 for 95%
    results = data[column_name].tolist()
    results.sort()
    lower_bound = int((1 - confidence_level) / 2 * len(results)) - 1
    upper_bound = int((confidence_level + 1) / 2 * len(results)) - 1
    if lower_bound < 0:
        lower_bound = 0
    return [round(float(results[lower_bound]), 1),
round(float(results[upper_bound]), 1)]


def summary_results(bootstrap, outfile, varx, val, gp):
    # INPUTS
    # "bootstrap": the bootstrap results imported as a dataframe (eg.
using pd.read_csv). precisely the output file from bootstrap_life_ex
    # "outfile": the name of the file to output the summary of the
bootstrap results to (extension included)
    nom_best = float(bootstrap[varx + ": " + gp + 'Nominee']['Best
Estimate'])
    win_best = float(bootstrap[varx + ": " + gp + 'Winner']['Best
Estimate'])
    con_best = float(bootstrap[varx + ": " + gp + 'Control']['Best
Estimate'])

    nomwin_best = round(win_best - nom_best, 1)
    wincon_best = round(win_best - con_best, 1)
    nomcon_best = round(nom_best - con_best, 1)

    boot_df = bootstrap.drop(['Best Estimate', 'Bootstrap Run'])
    boot_df.index.name = 'Bootstrap Run'

    nom_msm = confidence_interval(boot_df, varx + ": " + gp + 'Nominee',
0.95)
    win_msm = confidence_interval(boot_df, varx + ": " + gp + 'Winner',
0.95)
    con_msm = confidence_interval(boot_df, varx + ": " + gp + 'Control',
0.95)

    boot_df['Nominee Winner Difference'] = pd.to_numeric(boot_df[varx +
": " + gp + 'Winner'], errors='ignore') - pd.to_numeric(boot_df[varx + ":
" + gp + 'Nominee'], errors='ignore')
    boot_df['Control Winner Difference'] = pd.to_numeric(boot_df[varx +
": " + gp + 'Winner'], errors='ignore') - pd.to_numeric(boot_df[varx + ":
" + gp + 'Control'], errors='ignore')
```

```
    boot_df['Nominee Control Difference'] = pd.to_numeric(boot_df[varx +
": " + gp + 'Nominee'], errors='ignore') - pd.to_numeric(boot_df[varx +
": " + gp + 'Control'], errors='ignore')

    nomwindiff = confidence_interval(boot_df, 'Nominee Winner
Difference', 0.95)
    conwindiff = confidence_interval(boot_df, 'Control Winner
Difference', 0.95)
    nomcondiff = confidence_interval(boot_df, 'Nominee Control
Difference', 0.95)

    MSMPointEstimates = [win_best, nom_best, con_best, nomwin_best,
wincon_best, nomcon_best]
    MSMConfIntervals = [win_msm, nom_msm, con_msm, nomwindiff,
conwindiff, nomcondiff]

    MSM_column = []
    for i in range(0, len(MSMPointEstimates)):
        MSM_column.append(str(MSMPointEstimates[i]) + " " +
str(MSMConfIntervals[i]))

    index = ['Winner', 'Nominee', 'Control','Winner-Nominee Difference',
'Winner-Control Difference', 'Nominee-Control Difference']
    sum_df = pd.DataFrame(list(zip(MSM_column, index)),columns=[(varx +
": " + gp), 'Measure'])
    sum_df.set_index('Measure', inplace=True)
    sum_df.to_csv(outfile)
    return [sum_df]



def repeat(all_data, varx, val, gp):
    sub_df = all_data[all_data[varx] == val]
    bootstrap_life_ex(sub_df, states, statuses, 2,
varx+str(val)+"_"+gp+"_"+"LE.csv",varx, val, gp)
    boot_file = pd.read_csv(varx+str(val)+"_"+gp+"_"+"LE.csv", names =
[varx + ": " + gp + 'Nominee', varx + ": " + gp + 'Winner', varx + ": " +
gp + 'Control'])
    #df[varx + ": " + gp + 'Nominee', varx + ": " + gp + 'Winner', varx +
": " + gp + 'Control'] = boot_file
    summary_results(boot_file, varx+str(val)+"_"+gp+"_"+"Summary.csv",
varx, val, gp)
    #boot_file.columns=['a','b','c']
    #index = ['Winner Life Expectancy', 'Nominee Life Expectancy',
'Control Life Expectancy','Winner-Nominee Difference', 'Winner-Control
Difference', 'Nominee-Control Difference']
    #Summary = pd.DataFrame(list(zip(sumx, index)),columns=[(varx+ ": " +
gp), 'Measure'])
    #LE = pd.DataFrame(, columns=['Nominee Life Expectancy', 'Winner Life
Expectancy', 'Control Life Expectancy'])
    #LE.append(boot_file)


repeat(df, 'group', 1, 'gp1')
```

```
repeat(df, 'group', 2, 'gp2')
repeat(df, 'group', 3, 'gp3')
repeat(df, 'group', 4, 'gp4')

repeat(df, 'group', 5, 'gp5')
repeat(df, 'group', 6, 'gp6')
repeat(df, 'group', 7, 'gp7')
repeat(df, 'group', 8, 'gp8')
```

§4) SAS Codes for Validation Analysis

The attached document contains the single file with SAS codes using the proportional hazards model as a test of robustness. These models included a time-dependent step-function to account for age at first film, age at study enrolment, age at first nomination, and age at first win.

```
Options dkrOCond=error mergeNoBy=error msgLevel=i ;


libname data 'C:\Users\Default\Desktop\stars\data';


proc datasets lib=work kill;
quit;

ods html close;
ods listing;

*****************************************************************************;
* Importing csv dataset into SAS;
*****************************************************************************;

* fixed one data entry error - number of nominations from 11 to 1 in the CSV file;
PROC IMPORT OUT= WORK.stars DATAFILE=
"C:\Users\NEVET\Desktop\Stars\python\MoreForDeva.csv"
            DBMS=csv REPLACE;
     GETNAMES=YES;
RUN;

/*
proc means n nmiss data=stars;
      var First_:;
run;
*/

/*
proc freq data=stars;
      tables status;
run;
*/


data stars2;
      set stars;
      * deleting two stars who died just before becoming celebrities;
      if locator=337 then delete;
      if locator=1776 then delete;

      * changing status for a star who died just before winning;
      if locator=6085 then do;
            First_win=.;
            Status='Nominee';
            _Wins=.;
            MultiWinner=.;
      end;

      * calculating age at each state;
      age_of_first_movie = First_Movie - Birth;
      age_of_celebration = First_ceremony - Birth;
      age_of_nomination = First_nom - Birth;
      age_of_victory = First_Win - Birth;

      * Categorizing birth era;
      if birth<=1919 then BirthEra=0;
      else if birth>=1920 then BirthEra=1;
run ;
```

```
* Checking age at each state;
/*
proc means n nmiss data=stars2;
      var age_:;
      class status;
run;
*/

*********************************************************************************;
* Restructuring dataset so that there is a record for each transition per star;

*1);
* For controls, there are only 2 possible transitions:
Birth to celebrity
Celebrity to final;
data controls;
      set stars2;
      if status='Control';
      do i=1 to 2;
            if i=1 then do;
                  start       =0;
                  stop        =age_of_celebration;
                  performer   =0;
                  nominee           =0;
                  winner            =0;
                  endpt       =0;
                  state       =0;
                  output;
            end;
            else if i=2 then do;
                  start       =age_of_celebration;
                  stop        =Final - birth;
                  performer   =1;
                  nominee           =0;
                  winner            =0;
                  endpt       =1-alive;
                  state       =1;
                  output;
            end;
      end;
      drop i;
run;


*2);
* For nominees, there are 3 possible transitions:
Birth to celebrity
Celebrity to nominee
Nominee to final;

* if a star makes 2 transitions in the same year, adding 0.01 years to the latest state;
data nominees;
      set stars2;
      if status='Nominee';
      do i=1 to 3;
            if i=1 then do;
                  start       =0;
                  stop        =age_of_celebration;
                  performer   =0;
                  nominee           =0;
                  winner            =0;
                  endpt       =0;
                  state       =0;
```

```
				output;
			end;
			else if i=2 then do;
				start		=age_of_celebration;
				if age_of_nomination = age_of_celebration then
					stop	=age_of_nomination+0.01;
				else stop = age_of_nomination;
				performer	=1;
				nominee		=0;
				winner		=0;
				endpt		=0;
				state		=1;
				output;
			end;
			else if i=3 then do;
				if age_of_nomination = age_of_celebration then
					start =age_of_nomination+0.01;
				else start	= age_of_nomination;
				stop		=Final - birth;
				performer	=0;
				nominee		=1;
				winner		=0;
				endpt		=1-alive;
				state		=2;
				output;
			end;
		end;
		drop i;
run;

*3);
* For winners, there are 4 possible transitions:
Birth to celebrity
Celebrity to nominee
Nominee to winner
Winner to final;

* if a star makes 2 transitions in the same year, adding 0.01 years to the latest state;
data winners;
		set stars2;
		if status='Winner';
		do i=1 to 4;
			if i=1 then do;
				start		=0;
				stop		=age_of_celebration;
				performer	=0;
				nominee		=0;
				winner		=0;
				endpt		=0;
				state		=0;
				output;
			end;
			else if i=2 then do;
				start		=age_of_celebration;
				if age_of_nomination = age_of_celebration then
					stop	=age_of_nomination+0.01;
				else stop = age_of_nomination;
				performer	=1;
				nominee		=0;
				winner		=0;
				endpt		=0;
				state		=1;
				output;
```

```
			end;
			else if i=3 then do;

				if age_of_nomination = age_of_celebration then
					start =age_of_nomination+0.01;
				else start  = age_of_nomination;

				if age_of_nomination = age_of_victory then
					stop  =start+0.01;
				else stop  = age_of_victory;

				performer   =0;
				nominee           =1;
				winner            =0;
				endpt       =0;
				state       =2;
				output;
			end;
			else if i=4 then do;
				if age_of_nomination = age_of_victory then
					start =start+0.01;
				else start  = age_of_victory;
				stop        =Final - birth;
				performer   =0;
				nominee           =0;
				winner            =1;
				endpt       =1-alive;
				state       =3;
				output;
			end;
		end;
		drop i;
run;

********************************************************************************;

* adding all records back;
data stars3;
	set controls nominees winners;
	rename start = time1 stop=time2;
	drop alive;
run;

proc sort data=stars3;
	by locator time1;
run;

* we are interested only in 3 states: celebrities, nominees, and winners;
data stars4;
	set stars3;
	if time1 = time2 then time2=time2 + 0.01;
	if state in (1,2,3);
	survive=1-endpt;
run;


*ods pdf
file='C:\Users\NEVET\Desktop\Stars\data\output\stars_phreg_timedep_21Sep2020.pdf';

proc format;
	value ynf 1='Yes' 0='No';
	value eraf 0='Remote' 1='Recent';
	value statef 1='Control' 2='Nominee' 3='Winner';
```

```
run;

* Changing character variables to numeric variables;
data stars5;
      set stars4;

      Born_Whitex = (Born_White='Yes');
      Born_USAx = (Born_USA='Yes');
      Name_Changex = (Name_Change='Yes');
      Drama_Genrex = (Drama_Genre='Yes');

      format BirthEra eraf. Male_1 Born_Whitex Born_USAx Name_changex Drama_Genrex ynf.
state statef.;
run;

*********************************************************************************;
* Univariable COX regression models
*********************************************************************************;

option nodate;
ods output parameterestimates=a;
proc phreg data=stars5  covs(aggregate);
      class state(ref='Control' param=ref);
      model(time1 time2)*endpt(0)=state/rl ties=breslow;
      id locator;
run;

ods output parameterestimates=b;
proc phreg data=stars5  covs(aggregate);
      class BirthEra(ref='Remote' param=ref);
      model(time1 time2)*endpt(0)=BirthEra/rl ties=breslow;
      id locator;
run;

ods output parameterestimates=c;
proc phreg data=stars5  covs(aggregate);
      class Male_1(ref='No' param=ref);
      model(time1 time2)*endpt(0)=Male_1/rl ties=breslow;
      id locator;
run;

ods output parameterestimates=d;
proc phreg data=stars5  covs(aggregate);
      class Born_whitex(ref='No' param=ref);
      model(time1 time2)*endpt(0)=Born_whitex/rl ties=breslow;
      id locator;
run;

ods output parameterestimates=e;
proc phreg data=stars5  covs(aggregate);
      class Born_USAx(ref='No' param=ref);
      model(time1 time2)*endpt(0)=Born_USAx/rl ties=breslow;
      id locator;
run;

ods output parameterestimates=f;
proc phreg data=stars5  covs(aggregate);
      class Name_Changex(ref='No' param=ref);
      model(time1 time2)*endpt(0)=Name_Changex/rl ties=breslow;
      id locator;
run;

ods output parameterestimates=g;
```

```
proc phreg data=stars5 covs(aggregate);
      class Drama_Genrex(ref='No' param=ref);
      model(time1 time2)*endpt(0)=Drama_Genrex/rl ties=breslow;
      id locator;
run;

/*
ods output parameterestimates=h;
proc phreg data=stars5 covs(aggregate);
      model(time1 time2)*endpt(0)=age_of_first_movie/rl ties=breslow;
      id locator;
run;
*/

* Merge all univariable results together;
data UV;
      length parameter label $30;
      set a b c d e f g;
run;

ods html;
proc print data=UV; run;

*********************************************************************************;
* Multivariable COX regression models
*********************************************************************************;

* Without proportionality assumption testing;
ods output parameterestimates=i;
proc phreg data=stars5 covs(aggregate);
      class       state(ref='Control' param=ref)
                  BirthEra(ref='Remote' param=ref)
                  Male_1(ref='No' param=ref)
                  Born_whitex(ref='No' param=ref)
                  Born_USAx(ref='No' param=ref)
                  Name_Changex(ref='No' param=ref)
                  Drama_Genrex(ref='No' param=ref);
      model(time1 time2)*endpt(0)=state BirthEra Male_1 Born_Whitex Born_USAx
Name_Changex Drama_Genrex
                                                /rl ties=breslow;
      id locator;
run;

ods html;
proc print data=i;
run;

* With proportionality assumption testing for all covariates;
ods output parameterestimates=j;
proc phreg data=stars5 covs(aggregate);
      class       state(ref='Control' param=ref)
                  BirthEra(ref='Remote' param=ref)
                  Male_1(ref='No' param=ref)
                  Born_whitex(ref='No' param=ref)
                  Born_USAx(ref='No' param=ref)
                  Name_Changex(ref='No' param=ref)
                  Drama_Genrex(ref='No' param=ref);
      model(time1 time2)*endpt(0)=state BirthEra Male_1 Born_Whitex Born_USAx
Name_Changex Drama_Genrex
                                                BirthErat2 Male_1t2 Born_Whitext2
Born_USAxt2 Name_Changext2 Drama_Genrext2 /rl ties=breslow;
      id locator;
```

```
      Male_1t2 = Male_1 * log(time2);
      BirthErat2 = BirthEra * log(time2);
      Born_Whitext2 = Born_Whitex * log(time2);
      Born_USAxt2 = Born_USAx * log(time2);
      Name_Changext2 = Name_Changex * log(time2);
      Drama_Genrext2 = Drama_Genrex * log(time2);
      test_proportionality: test BirthErat2, Male_1t2, Born_Whitext2, Born_USAxt2,
Name_Changext2, Drama_Genrext2;
run;

ods html;
proc print data=j;
run;


* With proportionality assumption testing for birth era and country of birth;
ods output parameterestimates=k;
proc phreg data=stars5 covs(aggregate);
      class       state(ref='Control' param=ref)
                  BirthEra(ref='Remote' param=ref)
                  Male_1(ref='No' param=ref)
                  Born_whitex(ref='No' param=ref)
                  Born_USAx(ref='No' param=ref)
                  Name_Changex(ref='No' param=ref)
                  Drama_Genrex(ref='No' param=ref);
      model(time1 time2)*endpt(0)=state BirthEra Male_1 Born_Whitex Born_USAx
Name_Changex Drama_Genrex
                                                BirthErat2 Born_USAxt2 /rl ties=breslow;
      id locator;
      BirthErat2 = BirthEra * log(time2);
      Born_USAxt2 = Born_USAx * log(time2);
      test_proportionality: test BirthErat2, Born_USAxt2;

run;

ods html;
proc print data=k;
run;


* With proportionality assumption testing for country of birth;
ods output parameterestimates=l;
proc phreg data=stars5 covs(aggregate);
      class       state(ref='Control' param=ref)
                  BirthEra(ref='Remote' param=ref)
                  Male_1(ref='No' param=ref)
                  Born_whitex(ref='No' param=ref)
                  Born_USAx(ref='No' param=ref)
                  Name_Changex(ref='No' param=ref)
                  Drama_Genrex(ref='No' param=ref);
      model(time1 time2)*endpt(0)=state BirthEra Male_1 Born_Whitex Born_USAx
Name_Changex Drama_Genrex
                                                Born_USAxt2 /rl ties=breslow;
      id locator;
      BirthErat2 = BirthEra * log(time2);
      Born_USAxt2 = Born_USAx * log(time2);
      test_proportionality: test Born_USAxt2;

run;

ods html;
proc print data=l;
run; *ods pdf close;
```
